# Polyphenol metabolites in fermented foods: biotransformation, bioavailability, and functional roles

**DOI:** 10.3389/fnut.2026.1767453

**Published:** 2026-01-29

**Authors:** Nouf Abdullah Alharbi

**Affiliations:** Department of Basic Health Sciences, College of Applied Medical Sciences, Qassim University, Buraydah, Saudi Arabia

**Keywords:** bioavailability, fermentation, gut microbiota, microbial enzymes, polyphenols

## Abstract

Polyphenols, plant-derived bioactive compounds, are known for their antioxidant, anti-inflammatory, and antimicrobial properties, benefiting plant-based foods. Fermentation, driven by microbial enzymes like glycosidases, esterases, and decarboxylases, alters the chemical structure of polyphenols, enhancing their bioavailability and bioactivity. This review explores the transformation of polyphenols, particularly flavonoids and phenolic acids, during fermentation, resulting in bioactive metabolites with increased solubility, stability, and antioxidant activity, improving gastrointestinal absorption. Additionally, fermented polyphenol metabolites modulate gut microbiota by promoting beneficial bacteria such as *Lactobacillus* and *Bifidobacterium*, while inhibiting pathogens. These changes support gut health, reduce inflammation, and provide systemic benefits, including enhanced metabolic, immune, and neurocognitive functions. Despite progress, knowledge gaps remain, particularly regarding microbial pathways and the health outcomes linked to these metabolites. Future research should focus on mapping microbial biotransformation pathways of polyphenols and their impact on health outcomes. Additionally, well-controlled human intervention studies using multi-omics approaches are necessary to validate the systemic benefits of fermented polyphenol metabolites.

## Graphical Abstract

**Figure:**
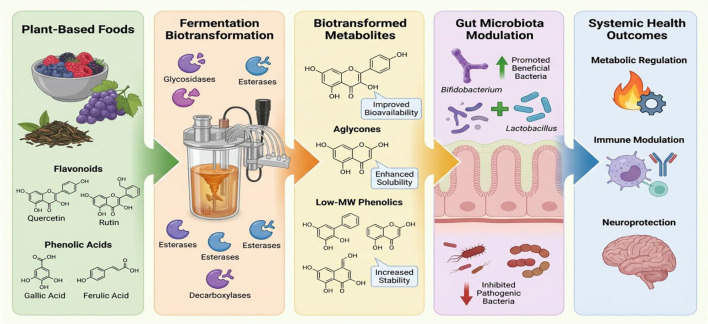

## 1 Introduction

Polyphenols are represented by a heterogeneous class of plant-derived secondary metabolites that form one of the most abundant groups of bioactive compounds in the human diet (1, 2). These compounds are widely distributed in a wide range of foods—including fruits, vegetables, cereals, legumes, cocoa, tea, coffee, wine and many other plant-based products—wherein they are responsible for various sensory properties, i.e., color, astringency, and overall organoleptic quality (3). Beyond their technological functions, dietary polyphenols have attracted a great deal of scientific attention because of their strong antioxidant, anti-inflammatory, antimicrobial, and immunomodulatory activity, which is increasingly linked to the prevention or modulation of chronic non-communicable diseases (4). Mechanistically, polyphenols may scavenge reactive oxygen species, affect redox-dependent signaling pathways, reduce pro-inflammatory mediators, and inhibit microbial cell structures and biofilm formation. This would make polyphenols ideal therapeutic agents for maintaining health as well as for managing disorders associated with inflammation (5). However, the *in vivo* biological effect of polyphenols is not only dependent on their intrinsic chemical structure, but also on their stability, solubility and transformation in the gastrointestinal tract with extensive metabolism by host and microbial enzymes yielding a broad spectrum of lower-molecular-weight metabolites often with markedly different bioactivity and bioavailability to the parent compounds (6).

With the better understanding of the biology of polyphenols, fermented foods have re-emerged as key players in functional nutrition and gut health research (7). The fermented foods, which include dairy products, fermented cereals, vegetables, legumes, cocoa, tea, coffee, and alcoholic beverages, contain complex microbial ecologies. Lactic acid bacteria (LAB), yeasts, and other microorganisms ferment the raw substrates into products with unique taste, nutritional profile, and function. These foods, once valued mainly for preservation, safety, and taste, are increasingly regarded nowadays as vectors of live microbes, their metabolites, and biotransformed food components capable of modulating the gut microbiome, enhancing the intestinal barrier integrity, influencing local and systemic immune responses, and impacting systemic metabolic functions (8). Clinical and preclinical studies have suggested that regular consumption of fermented foods is associated with enhanced gut microbial diversity, promotion of barrier integrity, and diminished systemic inflammation. Indeed, this provides a basis for their role as functional foods, particularly after more clear evidence of specific health benefit(s) may be established (9). In this broader context, the importance of fermentation as a modulator of phytochemicals such as polyphenols is increased as it can result in the generation of new or more bioavailable bioactive phytochemicals within the food matrix itself (10).

Such an association of polyphenols with fermentation can be envisaged as a two-way interaction between the plant compounds and the microbes involved. Resident microorganisms of foods produce a variety of enzymes during fermentation that are associated with polyphenols such as esters, glycosidases, decarboxylases, and reductases. These enzymes cleave glycosidic bonds, release phenolics bound to the cell wall, and induce changes in the structure of phenolic acids, flavonoids, and tannins (11). These biotransformations generally result in an increase in the percentage of free or low molecular weight phenolics, changes in conjugation patterns, improvements in solubility and may also enhance antioxidant and other functional properties as compared to the native polyphenol profile of the raw substrate (12). Meanwhile, certain polyphenols could selectively promote beneficial fermentative microorganisms while suppressing spoilage or pathogenic species, thereby impacting fermentation kinetics, microbial succession and final metabolite spectrum. Consequently, polyphenol rich fermented foods are often very different from their non fermented counterparts in qualitative and quantitative composition of phenolic compounds and their derived metabolites, and recent evidence indicates that these changes are associated with increased bioactivity in *in vitro* and *in vivo* systems (13).

Polyphenol metabolites formed during fermentation and subsequent passage through the gastro-intestinal tract are now considered to be important intermediaries in relation to the health benefits previously associated with polyphenol-rich and fermented foods. Following ingestion, phenolic metabolites derived from fermentation and some un-metabolized parent compounds are transported to the gut where they are further metabolized by colonic microbiota into small phenolic acids and lactone derivatives that have characteristic absorption abilities as well as biological targets (14). These metabolites are able to produce local effects on the gut environment by modulating the composition of the microbiota, inhibiting the growth of pathogenic bacteria, reducing biofilm formation and favoring the growth of beneficial taxa such as bifidobacteria, and through antioxidant, anti-inflammatory, metabolic and neuroprotective mechanisms (15). Recent studies demonstrate that fermentation may enhance these interactions by means of preconditioning polyphenol structures in the food matrix making them more accessible to host and gut bacterial enzymes, resulting in a higher pool of absorbable or microbiota-modulating metabolites present within the colonic lumen (16). Therefore, knowledge of how fermentation under specific conditions and microbials impacts on the polyphenol profile is important to properly design fermented functional foods in order to curtail gut health or inflammation-related diseases.

Despite the rapid advances, there are still gaps in the integration of polyphenol chemistry, fermentation science and nutrition. Most of the studies have looked at broad measures, such as total phenolics or overall antioxidant capacity, and detailed metabolite-by-metabolite analysis, clearly linked to specific health outcomes, are less common. The variation between analytical methodologies, microbial strains, fermentation parameters and raw materials also makes cross-study comparison more challenging and impedes the identification of meaningful structure and function relationships for fermentation-generated polyphenol metabolite production. Furthermore, gut microbiomes and metabolism are highly variable among individuals, and thus the *in vivo* fate and bioactivity of these metabolites can be very different from one person to another. That adds another layer to the hurdles when trying to translate lab or animal findings to human health guidance (17). To address these challenges, the systematic mapping of microbial biotransformation pathways, a quantitative metabolomics analysis of fermented food products and samples originating from biological samples are required alongside well-designed human intervention studies taking into account both aerial matrix and microbiome context (18).

In this review, we summarize recent findings about metabolites from polyphenols in fermented foods, with particular emphasis on the action of microbial enzymes and their functions as plant-derived raw materials. The chemical composition, biosynthetic routes and health-beneficial bioactivities of polyphenol metabolites with special emphasis on gut microbiota modulating potential, attenuation of oxidative stress and inflammation are discussed. The review uncovers their systemic effects in metabolic, immune and neurocognitive health. It also highlights knowledge gaps and makes recommendations on future prospective for polyphenolic-rich fermented foods and personalized dietary intervention.

## 2 Polyphenol chemistry

**Figure 1:**
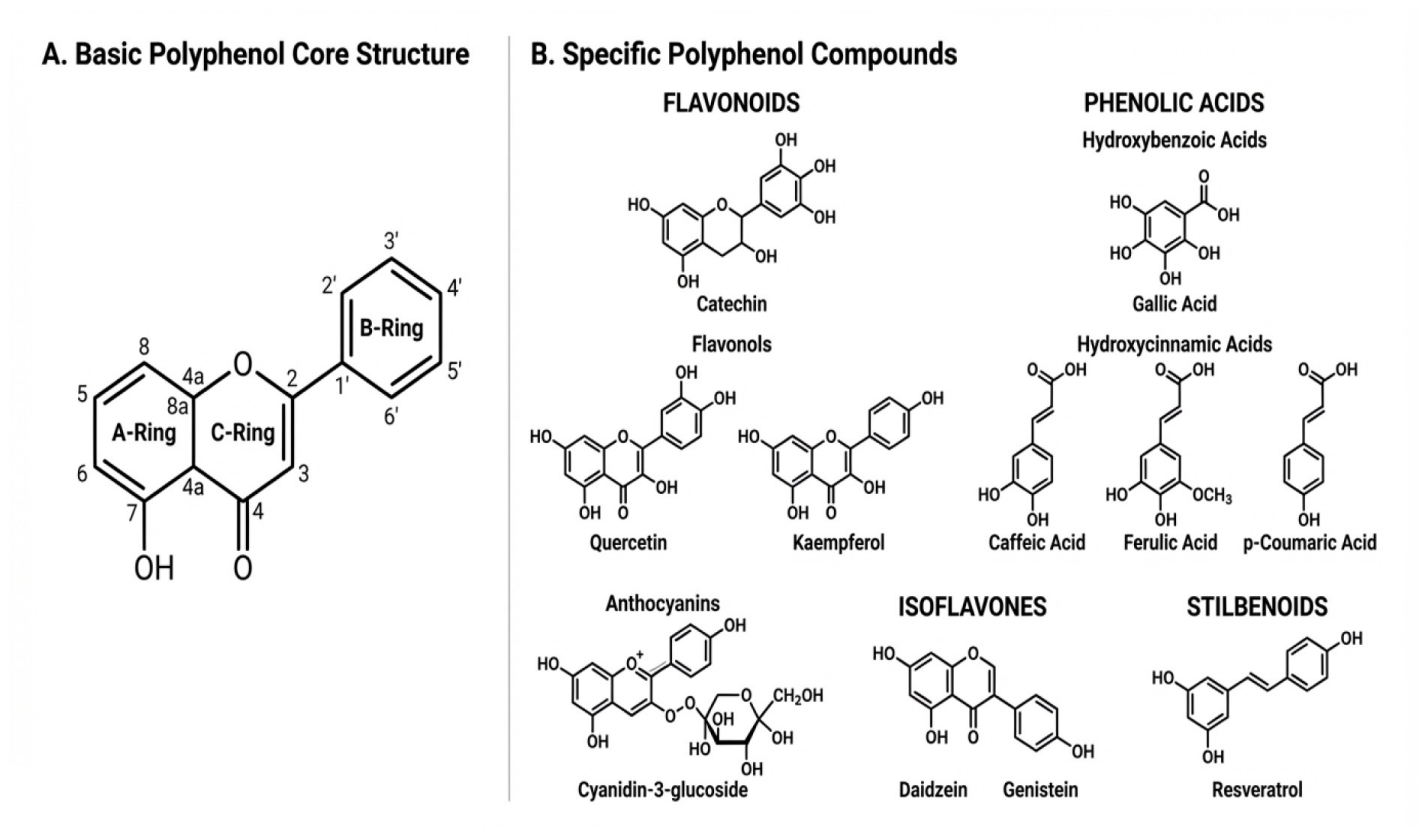
**(A)** Basic polyphenol core structure. **(B)** Different polyphenol compounds and their structure.

Polyphenols represent a large group of naturally occurring compounds in plants that are structurally defined by one or more phenolic rings as shown in Figure 1 (19). They are divided into a number of families according to share molecular scaffolds, which broadly tend to affect their chemical behavior and biological response. The main groups of dietary polyphenols are flavonoids and phenolic acids, which exist in a wide variety and are present in high amounts in fruits, vegetables and fermented diet (2).

All flavonoids possess a common structure consisting of two aromatic rings (A and B) linked together by a three-carbon bridge, which may frequently cyclize to form a heterocyclic ring (C-ring). This core architecture is modified in different ways giving rise to several subfamilies. It is interesting to note that the flavan-3-ols, such as catechins, do not have a double bond between carbon 2 and 3 and exhibit antioxidant properties (20). Flavonols such as quercetin and kaempferol contain a ketone moiety and a high-hydroxyl substituent, which play important roles in their chemical reactivity and biological activity. Anthocyanins are the glycosylated products of anthocyanidin, with sugars added to the molecules at selected positions affecting their solubility and stability (21).

Phenolic acids are simpler molecules, split mainly into hydroxybenzoic acids, such as gallic acid, and hydroxycinnamic acids which include caffeic and ferulic acids. They are frequently found as glycosidic or esterified conjugates with cell wall polysaccharides, participating not only in the structural integrity of plant tissues but also in their bioaccessibility and interactions during the digestion and fermentation processes (22). These polyphenolic moieties are constituted by functional groups as a pendent which stringently govern their chemical properties. Its capacity to donate electrons and neutralize free radicals is attributed mainly to the presence of hydroxyl (–OH) groups, which form the basis for its antioxidant properties. Methoxy (–OCH3) groups modify hydrophobicity and could increase metabolic stability, wherease glycosidic linkages to sugar reduce hydrophobicity, increase water solubility, and may have an impact on absorption/disposition in humans. These glycosides are often found in plant polyphenols with glucose, rhamnose or galactose as the most common carbohydrate moiety attached (23).

Polyphenols have different physicochemical properties ranging from highly water soluble phenolic acids to less soluble (larger) flavonoid polymers, depending on size, polarity and degree of polymerization. The stability of natural colors is influenced by factors such as pH, temperature, light—anthocyanins are extremely sensitive to pH and change color and stability in function thereof. The chemical reduction capacity of the polyphenolic structures creates their action as an antioxidant, which can serve by donating electrons to scavenge reactive oxygen species and reduce oxidative stress (24). Consequently, detailed chemistry of polyphenols, its core structures and functional group diversity that leads to their varied physicochemical behavior, is critically important in relation to such biological activities as well as transformation during fermentation and digestion. This chemistry help their reactions with enzymes, microbes and other components of food matrix and thus determines a range of health functions.

## 3 Sources and metabolism of polyphenols in fermented foods

Polyphenols are prevalent natural ingredients of many plant-based foods and have been used as basic materials in fermentations. The different polyphenol compounds and their dietry sources are summarized in Table 1. The variety of polyphenol-rich substrates significantly affects the quality and biofunction of fermented foods because these undergo biochemical changes at the molecular level during fermentation, resulting in both structural and functional modifications. In this regard, among the readily available raw materials such as grapes, legumes, soybean, different fruits have unique polyphenol profiles on which traditional and industrial fermentation systems can be based (25). Grapes have been found to be abundant in flavan-3-ols, anthocyanins, flavonols and phenolic acids mainly located in the skins and seeds. These compounds are at the core of wine's antioxidant potential and also to its sensorial properties (26). Soybeans also represent the main source of isoflavone glycosides and phenolic acids required for the nutritional and functional properties of fermented soy products such as soy sauce, miso, or tempeh. Fruit fermentations with berries, pomegranate and tropical fruits also utilize endogenous phenolic compounds such as flavonoids and phenolic acids for flavoring and associated health benefits (27).

**Table 1:** Sources of polyphenols in fermented foods.

**Compound class**	**Compound name**	**Dietary sources**	**Fermented food sources**	**Bioactive metabolites**	**References**
Flavan-3-ols	Catechin	Grapes, tea, cocoa, berries, apples	Red wine, white wine, kombucha, pu-erh tea, fermented cocoa	Epicatechin gallate, 3-O-methylcatechin, catechin sulfates	(34)
Flavan-3-ols	Epicatechin	Cocoa, grapes, tea, apples, broad beans	Red wine, dark chocolate, kombucha, fermented cocoa products	Epicatechin-3-O-gallate (EGCG), 3-O-methylepicatechin, epicatechin metabolites	(35)
Flavan-3-ols	Epicatechin	Cocoa, grapes, tea, apples, broad beans	Red wine, dark chocolate, kombucha, fermented cocoa products	Epicatechin-3-O-gallate (EGCG), 3-O-methylepicatechin, epicatechin metabolites	(36)
Flavan-3-ols	Procyanidin B2	Grapes, apples, cocoa, cranberries	Red wine, cider, fermented cocoa, craft beer	Procyanidin dimers, catechin, epicatechin	(37)
Anthocyanins	Cyanidin-3-glucoside	Berries (blackberries, raspberries), cherries, black rice	Red wine, fermented berry products, berry vinegar, fruit kombucha	Protocatechuic acid, phloroglucinaldehyde, cyanidin aglycone	(38)
Anthocyanins	Delphinidin-3-glucoside	Blueberries, blackcurrants, eggplant, pomegranate	Red wine, fermented blueberry juice, berry kefir	Gallic acid, syringic acid, delphinidin aglycone	(39)
Anthocyanins	Malvidin-3-glucoside	Red grapes, blueberries, bilberries	Red wine (major anthocyanin), fermented grape juice	Syringic acid, gallic acid, malvidin-3-O-glucuronide	(40)
Anthocyanins	Peonidin-3-glucoside	Cherries, cranberries, plums, red grapes	Red wine, fermented cranberry products, cherry wine	Vanillic acid, protocatechuic acid, peonidin aglycone	(41)
Flavonols	Quercetin	Onions, apples, berries, kale, broccoli	Red wine, kombucha, kimchi, sauerkraut, fermented onions	Quercetin-3-glucuronide, isorhamnetin, tamarixetin, 3,4-dihydroxyphenylacetic acid	(42)
Flavonols	Kaempferol	Kale, spinach, broccoli, tea, beans	Kombucha, fermented tea, kimchi, fermented vegetables	Kaempferol-3-O-glucuronide, kaempferide, kaempferol sulfates	(43)
Flavonols	Kaempferol	Kale, spinach, broccoli, tea, beans	Kombucha, fermented tea, kimchi, fermented vegetables	Kaempferol-3-O-glucuronide, kaempferide, kaempferol sulfates	(44)
Flavonols	Myricetin	Berries, grapes, wine, tea, vegetables	Red wine, kombucha, fermented berry products	Myricetin-3-O-glucuronide, syringetin, laricitrin	(45)
Phenolic Acids	Caffeic acid	Coffee, fruits, vegetables, herbs	Wine, kombucha, coffee (fermented), fermented vegetables, kimchi	Dihydrocaffeic acid, caffeic acid-3-O-glucuronide, caffeic acid sulfates	(46)
Phenolic Acids	Ferulic acid	Whole grains, coffee, vegetables, fruits	Miso, tempeh, soy sauce, fermented rice, sourdough bread, kombucha	Dihydroferulic acid, ferulic acid-4-O-sulfate, vanillic acid.	(47)
Phenolic Acids	p-Coumaric acid	Tomatoes, carrots, grains, peanuts	Wine, kombucha, fermented vegetables, miso, tempeh	Dihydro-p-coumaric acid, p-coumaric acid sulfates, 3-(4-hydroxyphenyl)propionic acid	(48, 49)
Phenolic Acids	Gallic acid	Tea, grapes, berries, nuts	Wine, kombucha, fermented tea, fermented fruits	Pyrogallol, 4-O-methylgallic acid, gallic acid sulfates	(50)
Phenolic Acids	Protocatechuic acid	Grapes, berries, olives, nuts	Wine, vinegar, fermented olives, kombucha	Protocatechuic acid-4-O-sulfate, 3,4-dihydroxyphenylacetic acid	(51)
Isoflavones	Daidzein	Soybeans, soy products, legumes	Tempeh, miso, natto, soy sauce, fermented tofu, doenjang	Equol, O-desmethylangolensin (O-DMA), dihydrodaidzein	(52)
Isoflavones	Genistein	Soybeans, soy products, legumes, fava beans	Tempeh, miso, natto, soy sauce, fermented soybeans	6-hydroxygenistein, 3-hydroxygenistein, genistein-7-O-glucuronide	(53)
Isoflavones	Glycitein	Soybeans, soy products, legumes	Tempeh, miso, natto, fermented soy products	6-hydroxydaidzein, glycitein-7-O-glucuronide, 6-methoxyequol	(54)
Proanthocyanidins	Procyanidin oligomers	Grapes, apples, cocoa, cranberries	Red wine, cider, fermented cocoa, grape seed extract products	Epicatechin, catechin, phenylvalerolactones, phenylpropionic acids	(55)
Proanthocyanidins	Theaflavins	Black tea	Fermented black tea, kombucha (black tea-based), pu-erh tea	Theaflavin-3-gallate, theaflavin-3-prime-gallate, gallic acid	(56)
Proanthocyanidins	Theabrownins	Pu-erh tea (fermented tea)	Pu-erh tea, aged fermented teas, dark tea	Gallic acid, catechin degradation products, quinones	(57)
Stilbenoids	Resveratrol	Grapes, berries, peanuts, pistachios	Red wine, fermented grape products, peanut-based fermented foods	Dihydroresveratrol, resveratrol-3-O-glucuronide, resveratrol sulfates, lunularin	(49)
Stilbenoids	Piceid	Grapes, grape juice, wine	Red wine, white wine, fermented grape juice	Resveratrol (via hydrolysis), piceid sulfates, piceid glucuronides	(58)
Stilbenoids	Pterostilbene	Blueberries, grapes, almonds	Blueberry wine, fermented berry products	Pterostilbene sulfates, pterostilbene glucuronides, pinostilbene	(59)
Ellagitannins	Ellagic acid	Pomegranate, berries (raspberries, strawberries), walnuts	Fermented pomegranate juice, berry wine, fermented berry products	Urolithin A, Urolithin B, Urolithin C, Urolithin D (gut microbiota-dependent)	(60)
Lignans	Secoisolariciresinol	Flaxseeds, sesame seeds, whole grains	Sourdough bread, fermented whole grain products, miso	Enterolactone, enterodiol (gut microbiota-dependent conversion)	(61)
Hydroxycinnamates	Chlorogenic acid	Coffee, tea, fruits, vegetables	Fermented coffee, kombucha, wine	Caffeic acid, quinic acid, dihydrocaffeic acid, ferulic acid	(62)
Flavanones	Hesperidin	Citrus fruits (oranges, lemons, grapefruits)	Fermented citrus products, citrus kombucha, yuzu fermented products	Hesperetin, hesperetin-7-O-glucuronide, hesperetin sulfates	(63)
Methylxanthines	Caffeine	Coffee, tea, cocoa, guarana	Kombucha, fermented coffee, fermented tea	Paraxanthine, theobromine, theophylline, 1-methylxanthine	(64)
Methylxanthines	Theobromine	Cocoa, chocolate, tea	Fermented cocoa, dark chocolate, kombucha (cocoa-based)	7-methylxanthine, 3-methylxanthine, xanthine	(65)

Through fermentation, the microbial enzymes are mainly responsible for biotransforming the polyphenols which in turn changes their chemical structure and increases their bioactivity. The critical enzymatic reactions involve the hydrolysis of glycosidic bonds by microbial β-glucosidase and other glycosidases, leading to the release of polyphenol aglycones which are more lipophilic and bioactive moieties (28). This transformation is of particular interest because numerous plant polyphenols are present conjugated to sugars, which in turn hinders their absorption in the gastrointestinal tract Fermentation increases the availability and activity of polyphenols through the cleavage of sugar moieties. Other microorganisms' enzymes, including tannases and esterases, play a role in the release of phenolic acids from their esterified forms as well as the depolymerization of tannins to enhance the free phenolics and low-molecular-weight compounds. The cooperative action of these enzymes not only releases bound polyphenols but can also strongly modify their antioxidant activity and interaction with the gut microbiota (11).

Fermentation also leads to the production of multiple, metabolic-pathway polyphenol metabolites. In addition to direct hydrolysis, other complex transformations like decarboxylation, ring cleavage, reduction, methylation and dehydroxylation take place during fermentation and give rise to new phenolic acid and flavonoid derivatives having different biological and sensory properties (29). For example, proanthocyanidins and polymeric flavonoids in raw materials can be degraded into small phenolic acids such as caffeic acid, ferulic acid and p-coumaric acid with increased bioavailability and antioxidant properties. Likewise, flavonoid aglycones produced during fermentation may get modified that make them more potent in modulating oxidative stress and inflammatory pathways of the human gut. These metabolites are generally found at higher concentrations in fermented by-products compared to their unfermented counterparts, indicating fermentation as a bio-enhancing processing step (30).

Various types of polyphenolic compounds, such as phenolic acids, flavonoids and stilbenoids are subjected to different microbial conversions during fermentation, mediated by certain microbial groups i.e., lactic acid bacteria (LAB) and yeasts. LAB species are known to be the major producers of β-glucosidase that is important in hydrolysis of isoflavone glycosides in legumes into more biologically active aglycone forms (31). The higher level of daidzein and genistein resulting from the enzymatic action during soy fermentation may increase the anti-inflammatory and antioxidant capacities of soy products. Demethylation and ring cleavage reactions are also performed by yeast, providing other enzymatic activities that transform flavonoids to create both sensory and bioactives in fermented beverages such as wine or kombucha. Stilbenoids such as resveratrol, despite their low content, may also be released or formed during fermentation that contribute to the functional variety of fermented foods (32). This interaction of microbial enzymes to polyphenolic substrates is strain dependant and also dependent on fermentation conditions such as pH, temperature and time of fermentation which makes it possible to produce desired metabolite profiles by tailored production (33).

In conclusion, the origins and metabolism of polyphenols in fermented foods illustrate a complex biochemical interplay between plant-derived compounds and microbial enzymatic machineries. More diverse starting materials such as grapes, soybeans, and fruits rich in polyphenols can also be used as substrates within this host, microbial fermentation itself causes structural changes by enzymatic hydrolysis and the generation of new metabolites. These conversion improve polyphenol bioaccessibility, change the antioxidant and anti-inflammatory effect, and generate metabolites with novel potential health benefits. Insight into these processes also offers a scientific foundation for optimizing fermentation approaches toward the production of functional foods that are rich in bioactive polyphenolic metabolites with known health benefits.

## 4 Bioavailability and bioactivity of fermented polyphenol metabolites

Bioavailability and bioactivity of polyphenols are key determinants of their health benefits, but the majority of naturally occurring dietary polyphenols exhibit low bioavailability, extensive metabolism, and rapid excretion. Fermentation has been increasingly recognized as a promising method to circumvent these drawbacks via enzymatic transformation of polyphenols into more soluble, stable and bioaccessible forms in addition to leading to the generation of new metabolic products harboring unique biological properties. Therefore, fermented foods and beverages are often reported to contain higher amounts of bioaccessible phenolic compounds than non-fermented products derived from similar raw materials (66).

### 4.1 Improved bioavailability via fermentation

One of the main processes with which fermentation enhances polyphenol bioavailability is to release conjugated forms into free aglycones and low-molecular-weight derivatives. In plant tissues, flavonoids and isoflavones are largely present as glycosides or esterified molecules, that show low intestinal permeability. Through fermentation, microbial β-glucosidases, esterases and tannase hydrolyse glycosidic and ester linkages resulting in aglycones and de-esterified phenolic acids with higher lipophilicity and better capacity for passive-diffusion or carrier-mediated absorption in the small intestine (67). For instance fermentation of tea, cocoa and fruit-based substrates has been found to enhance the levels of flavonoid aglycones and/or simple phenolic acids *in vitro* which also translates into improved *in vitro* bioaccessibility as well as higher plasma concentrations of these metabolic products in animal or human time interventions. Soy-derived isoflavone glycosides are converted into aglycones such as daidzein and genistein when soy-based products are subject to fermentation, thus having higher absorption and greater estrogenic activity together with antioxidant action, highlighting how fermentation can “release” bioactive activity from these class of polyphenols (68).

Fermentation also affects physicochemical characteristics, favoring bioavailability such as solubility and stability. The microbial metabolism may produce more polar or hydrophilic derivatives which enhance solubility in aqueous intestinal environment, Or smaller and less complex molecules that are less likely to precipitate and have a more favorable interaction with transport systems (69). Furthermore, fermentation might also contribute to stability by converting very labile molecules into less labile metabolites in the face of pH-related degradation, enzymatic oxidation or thermal degradation and thus increase how much of the parent polyphenol material is left after processing and gastric transit. This combined effect of deglycosylation, depolymerization and stabilization causes a higher bioavailability of phenolics and their metabolites in fermented foods and beverages (70).

### 4.2 Biological activities of fermented polyphenol metabolites

Polyphenol metabolites originating from fermentation can have several biological activities once absorbed or present in the gut lumen. All of these activities are either increased or qualitatively different than the non-fermented material, due to structural changes leading to modified redox properties, receptor interactions and microbial targets. The standard reductions and oxidation potentials and chemical accessibility of hydroxyl moieties that can facilitate the free radical scavenging ability of fermented polyphenol metabolites are improved. Deglycosylation and depolymerization may allow for the exposure of more phenolic hydroxyls or for production of smaller phenolic acids with radical scavenging capability, leading to stronger DPPH neutralizing power. In fermented teas, cocoa products and fruit wines the inhibition of lipid peroxidation and cellular antioxidant response has been demonstrated to be superior to unfermented controls while being associated with increased presence of fermentation-derived phenolic acids and flavonoid aglycones (71).

Once absorbed or active in the gut lumen, fermentation-derived polyphenol metabolites exert a range of biological activities. Many of these activities are enhanced or qualitatively different compared with non-fermented forms because structural modifications alter redox properties, receptor interactions, and microbial targets (72). Fermented polyphenol metabolites usually exhibit higher antioxidant power in chemical and cellular systems as a result of enhanced redox potential and accessibility of hydroxyl groups. After deglycosylation and depolymerizaiion, phenolic hydroxyls are exposed and neutralize reactive oxygen species and reactive nitrogen species effectively (73). Antioxidant capacity and inhibition of lipid peroxidation, has also been proved to be higher with fermented teas (theaflavins), cocoa products (polyphenols) and fruit wines than unfermented controls which had been attributed to the accumulation of phenolic acids and flavonoid aglycones derived from fermentation (71).

Fermented polyphenols and their metabolites can inhibit the inflammatory response by regulating the expression of several proteins involved in signaling pathways, including NF-κB, MAPKs and cytokine production. Fermentation-induced structural changes would enable improved bindings to the transcription factors or enzymes related to inflammation, leading in turn to downregulation of pro-inflammatory markers such as TNF-α, IL-6, and COX-2 (5). Fermented plant extracts, mainly from soy, cereals or fruits, are more potent in inhibiting inflammatory markers than non-fermented counterparts which is explained by higher levels of certain aglycones and low molecular weight phenolic acids resulting due to fermentation (74). Fermentation-mediated conversions may enhance the anticancer activity of pulse and cereal polyphenolics through production of metabolites with enhanced cellular uptake as well as enhancing effect in relation to cell proliferation and apoptosis. Fermented extracts of pulses are known to inhibit growth of cancer cells, induce cell cycle arrest and apoptosis at a lower concentration than the unfermented extract, thus possessing better anticancer activity (75). Mechanistically, these actions consist of the regulation of oxidative stress, disturbance in growth factor signaling and activation of intrinsic apoptotic pathways, where fermentation metabolites such as phenolic acids and flavonoids derivatives are shown to play a crucial role (76).

In the colon, fermentation derived polyphenol metabolites closely interact with gut microbiota functioning as substrates or effectors that modulate microbial composition and activity. Several low molecular weight phenolic acids produced during fermentation have demonstrated selective inhibition of potential pathogenic bacteria and support growth of some beneficial microoraganism such as *Lactobacillus* and *Bifidobacterium*, suggesting a prebiotic-like modulation of the microbiome (77). Additionally, fermentation increases phenolics content that support gut barrier integrity, while preventing oxidative stress in the intestinal mucosa and reducing inflammatory signals derived from the gut (78). All these effects contribute to maintain gut homeostasis and can reduce the risk of diseases like inflammatory bowel disease or metabolic endotoxemia.

Fermented polyphenol containing products may have a beneficial effect on cardiovascular and metabolic markers, as well as blood pressure, lipid profiles, and insulin sensitivity. Higher bioavailability of certain fermentation-derived metabolites has been linked to enhanced endothelial function, lower oxidative modification of LDL, and reduced levels of systemic inflammatory markers (79). Among metabolic studies, consumption of fermented foods with high levels of bioactive phenolic metabolites has been inversely associated with glucose homoeostasis and features of the metabolic syndrome in part as a result of antioxidant, anti-inflammatory and microbiota-mediated action (80). Altogether, fermentation converts polyphenols from relatively inactive or low bioavailable species to a tissue-active library of uniquely metabolized and highly diverse array of metabolites with the highest bioavailability profile and extensive array of bioactivities. Based on the optimization of fermentation conditions, responsible microbial strains and available polyphenol-rich substrates, functional fermented foods can be designed to increase their levels in beneficial metabolites for antioxidant defense, anti-inflammatory activities, gut and cardiometabolic health protection as well as potentially a decrease in cancer risk.

## 5 Microbial mechanisms in polyphenol biotransformation

Microbial consortia are the essence of polyphenol bio-transformation in fermented foods by converting complex plant phenolics into structurally diverse metabolites that vary in availability and bioactivity. During fermentation, bacteria, yeasts and molds produce an array of enzymes that hydrolyze, oxidize, reduce and rearrange the structures of polyphenols with LAB exhibiting a particularly significant effect (81). These microbial activities not only determine the nutritional and functional characteristics of fermented products but also affect their sensory attributes and microbial stability, rendering polyphenol metabolism an important interface between food chemistry and fermentation microbiology (9).

### 5.1 Role of lactic acid bacteria (LAB)

Lactic acid bacteria (LAB) are the most common starter cultures and characterize several natural fermentations of vegetables, cereals, legumes and some beverages in which they play a key role in polyphenol biotransformation. They are also known to produce glycosidases such as β-glucosidase, α-rhamnosidase, esterases and tannases which catalyze the release of flavonoid and phenolic acid conjugates from their bound forms into aglycones, or simpler phenolics with augmented biological activity (82). *Lactiplantibacillus plantarum* formerly known as *Lactobacillus plantarum* is amongst the model species commonly found in wine, olive, vegetable and cereal fermentations and acknowledged for its polyphenol rich environment tolerance, as well as versatile enzymatic arsenal. *L. plantarum* is able to hydrolyze flavonoid glycosides, release phenolic acids from esterlinked forms and further convert the hydroxycinnamic acid via decarboxylation and reduction producing derivatives with changed antioxidant and antimicrobial activities (83).

Liu et al. (84) reported that the sensory quality and acceptability of Aronia melanocarpa juice fermented with *Lactobacillus plantarum 1243* were enhanced. Metabolic changes improve the release of phenolic acids, anthocyanins and flavonoids but not the antioxidizing activity. Liu et al., (85) found that LAB fermentation of Eucommia ulmoides tea affected the physicochemical properties, antioxidant activity, and aroma. *Lactobacillus bulgaricus* stimulated chlorogenic and geniposidic acids at greater antioxidant potential compared with the other strains. It also contributed to the fruit and floral flavor notes, once again revealing strain-dependent effects on functional tea quality. This research provides further confirmation that LAB-mediated enzymatic hydrolysis is a major factor in transforming glycosylated and cell wall-bound polyphenols into compounds with enhanced bioactivity. LAB can also be involved in ring-fission and reductive transformation of flavonoids and phenolic acids, also but these pathways are mapped only fragmentarily so far and they seem to be strongly strain-dependent. Direct enzymatic and indirect changes in pHand redoxconditions, LAB effectively re-modeling the migrating polyphenolic landscape during food fermentation (11).

### 5.2 Importance of other microbes

Non-LAB microorganisms, especially yeasts and molds, are also critical contributors to polyphenol biotransformation in the process of fermentation, frequently cooperating with LAB for the generation of complex metabolite patterns. Yeasts such as *Saccharomyces cerevisiae* and different non-Saccharomyces species are the main actors of alcoholic fermentations of wine, beer, cider, kombucha in which glycosidases, oxidoreductases, lyases acting on phenolics exist. These enzymes are able to de-glycosylate flavonols and anthocyanins, as well as oxidize or reduce phenolic structures, and produce volatile phenolic compounds that have an impact on aroma and flavor (86). Yeast-mediated fermentations of cocoa and coffee modify the oxidation state and polymerization of endogenous polyphenols, resulting in modifications of astringency, bitterness, antioxidant activity and shelf stability. Thus by selectively modifying phenolic molecules, yeast metabolism contributes to sensory development as well as to the functional attributes of fermented products (87).

**Figure 2:**
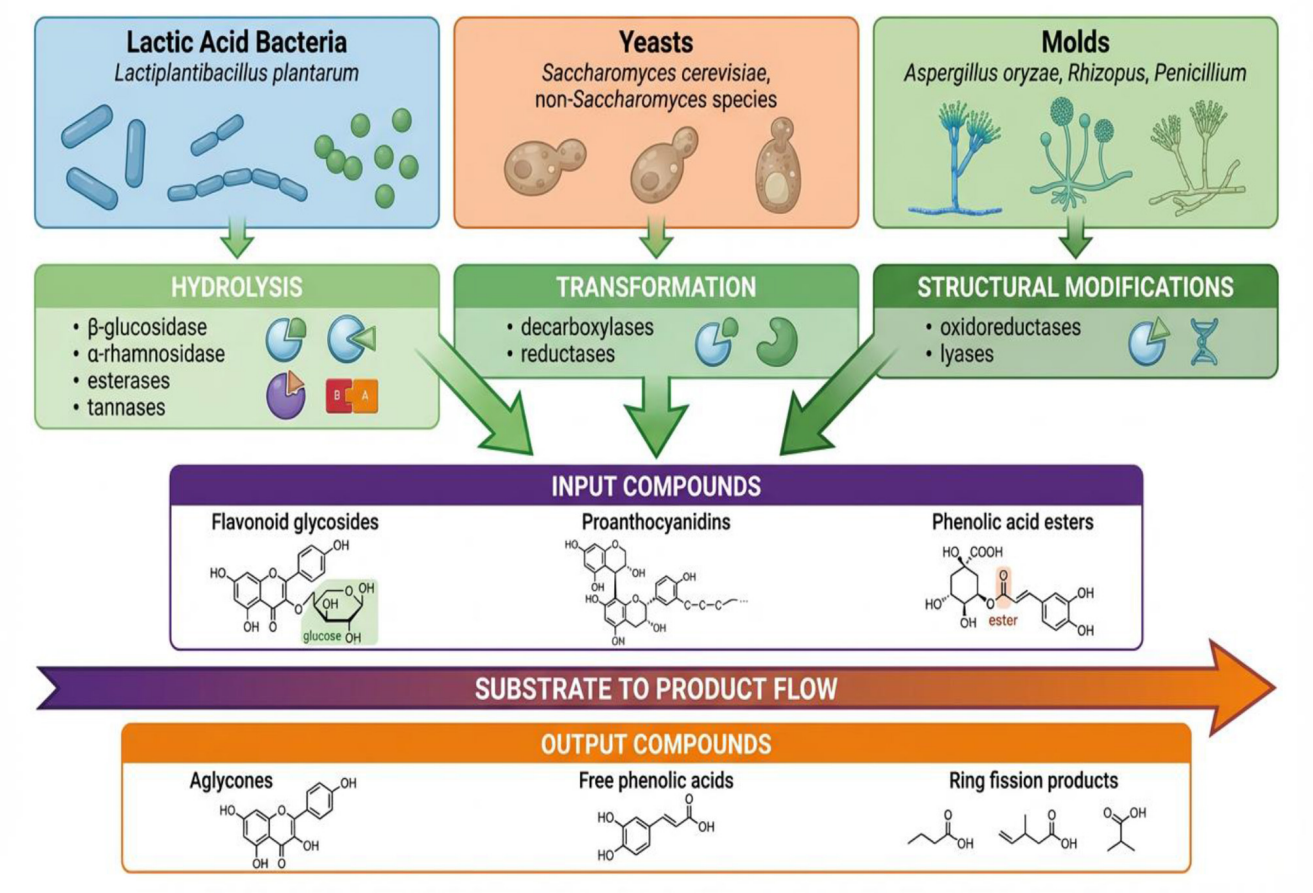
Microbial mechanisms in polyphenol biotransformation.

Molds such as Aspergillus, Rhizopus and Penicillium are particularly important in fermentations like miso, soy sauce tempeh and some types of cheeses, where the produce a vast range of extracellular enzymes that degrade plant cell wall matrices to release bound phenolic compounds as shown in Figure 2. Furthermore, koji molds like *Aspergillus oryzae* are source of β-glucosid, tannase, and feruloyl esterase activities releasing isoflavone aglycones and phenolic acids from soy and cereal-based source to enhance the bioactive compounds concentration along with flavor complexity development (88). Co-cultivation of LAB with enological yeasts improves functional and flavor parameters of multi-substrate beverages (89). *Pichia kluyveri* PK7 could enhance fermentation, amino acid level and even hypolipidemic effect. *Kluyveromyces marxianus* KM20 enhanced the antioxidant ability and ester production, highlighting that strain-specific synergistic effects of co-fermentation. These non-LAB microbes, together with LAB, constitute a functional consortium, which ultimately determines the composition of the final polyphenol metabolites found in fermented foods.

### 5.3 Integration of multi-omics methods

Multi-omics strategies offer a strong system to unravel the intricate webs of microbial community elements that convert polyphenols during fermentations, enabling to connect “who is there” with “what they are doing” and “which metabolites are formed”. Rather than looking at single enzymes or a few strains in isolation, multi-omics includes metagenomics, metatranscriptomics, proteomics and metabolomics to create fermentation as a dynamic system within many layers. This systems view, is especially relevant for polyphenols, as these molecules are a structurally diverse group that occurs in various conjugated forms and undergoes sequential or cooperative metabolism by distinct microbes (90).

Metagenomics provides the description of the taxonomic and functional gene inventory of the fermentation microbiome. By total DNA sequencing of the fermenting matrix, it is possible to identify dominant as well as subdominant taxa of fermentation microorganism such as Lactiplantibacillus spp., Leuconostoc spp., Saccharomyces spp, Aspergillus. The gene annotation may reveal genes encoding enzymes in polyphenol metabolism including β-glucosidases, α-rhamnosidases, esterases, tannin acylhydrolases tannases decarboxylase, dehydrogenase and reductase. Comparative metagenomics among different time points or fermentation conditions provides indications of variations in community structures and relative abundance of these functional genes, and can thus suggest which organisms may have the capacity to lead specific steps of polyphenols biotransformation. Meanwhile, metagenomics cannot by itself reveal whether these genes are active, so it is typically paired with expression-level data (91). Metatranscriptomics fills this gap by sequencing community RNA to assess which genes are transcribed during individual fermentation states. By quantification of transcripts of genes for expression of β-glucosidase or phenolic acid decarboxylase can detect the changes in the phenolic profiles. This is particularly beneficial in mixed fermentations where multiple strains may possess the same genes, metatranscriptomic data can indicate which taxa are active with respect to transcription under particular conditions (92).

Proteomics goes one step further by the direct quantification of proteins and enzymes, thereby brid ging between gene expression and catalytic activity. Shotgun or targeted proteomics can help to reveal the precise enzyme machinery participating in polyphenol catabolism, including particular β-glucosidase isoforms, tannases, feruloyl esterases or dehydrogenases that play a role at different time points during fermentation or when using distinct fermentation conditions. Post-transcriptional regulation, protein stability and enzyme secretion can all affect functional output, so that proteomic data frequently refines or counters conclusions based on transcript-level analysis alone (93). Functional metagenomic screening combined with glycoanalytics resulted into the identification of enzymes that cleaved sulfated N-glycans. xCGE-LIF enabled high-resolution glycomic profiling, confirming that the enzyme is specific for GlcNAc-6-SO4 modifications. This multi-omic approach shows the improvements in analyses of structure and function of the glycans which can be made when genomics is combined with glycomics (94).

Metabolomics specifically collects the fermentation-induced alterations of polyphenols and their metabolites. Metabolomics with LC–MS/MS, UHPLC–HRMS or NMR-based platforms profiles the parent polyphenols such as flavonoid glycosides, proanthocyanidins and phenolic acid esters, as well as intermediates and end products of metabolism including free phenolic acids, aglycone flavonoids, ring-fission products and small aromatic acids. Time-resolved metabolomics allows to map transformation pathways by tracking the disappearance of substrates and the occurrence of product alongside, in many cases, multi-step events populated by distinct element groups (95). Taken together, the integration of metagenomics, metatranscriptomics, proteomics, and metabolomics provides a mechanistic picture on how fermentation systems process complex polyphenol mixtures into specific bioactive metabolite profiles and how these changes are linked to microbial community dynamics. This understanding allows for the rational development of starter cultures, co-cultures, and process parameters through control of polyphenol biotransformation into targeted metabolites that have potential beneficial effects on fermented food quality and human health.

## 6 Functional roles of polyphenol metabolites in gut health

### 6.1 Gut microbial modulation

Polyphenol metabolites generated by food fermentation and further gut microbiota metabolism become key effectors modulating the intestinal ecosystem through impact on microbial composition and host inflammatory conditions. These metabolites can also function as selective growth factors for commensal to help and maintain health of the gut and provide systemic benefits (96). Polyphenol-metabolites mediated fermentation acts directly within the gut to exert selective pressures on enteric microbiota, as commensals and pathogens differ substantially in their capacity to use such metabolites as substrates or be antimicrobially impacted by them. Individual, small phenolic acids and flavonoid catabolites can be utilized by beneficial genera such as *Bifidobacterium* and *Lactobacillus* to support their growth and metabolic activity, while higher local concentrations of particular phenolics inhibit pathogenic organisms such as Clostridioides difficile, Escherichia coli or other Enterobacteriaceae (97). Post fermentation phenolic plant metabolites promoted increased populations of *Lactobacillus* and Bifidobacterium in experimental models, while reducing pro inflammatory and proteolytic taxa involved in dysbiosis (98). Also, fermented polyphenol extracts increased the population of beneficial LAB and bifidobacteria in the colon as well as reduced the populations of inflammatory and endotoxin-producing microorganisms and exert prebiotic-like effect (99). These observations showed that polyphenol metabolites contribute to influence a particular microbiota profile toward one more favorable for barrier function, short chain fatty acid production and decreases susceptibility to infection.

### 6.2 Reduction of gut inflammation

Polyphenol-derived metabolites also aid in reducing gut inflammation by acting directly on host cells and indirectly through the modulation of microbiota. The fermented and microbiota-derived phenolic acids possess anti-inflammatory effects, such as inhibiting NF-κB activation, reducing pro-inflammatory cytokines likes TNF-α, IL-1β, IL-6 expression and increasing the synthesis of anti-inflammatory mediators like IL-10 on intestinal epithelial cells and immune cells (100). Polyphenol-rich substrates fermented by LAB-derived metabolites that dampen the expression of inflammatory markers and oxidative stress in intestinal cell models and experimental colitis were associated with simultaneous increased levels of beneficial and decreased levels of pro-inflammatory bacteria (101). Li et al. (133) has also reported that certain microbial polyphenol metabolites could attenuate aspects of inflammatory bowel disease (IBD) in pre-clinical models by strengthening the tight junction, decreasing mucosal oxidative damage, and modulating of the microbial community toward a more anti-inflammatory profile. By such pathways, fermented food-derived polyphenol metabolites may alleviate chronic low-grade gut inflammation and as adjuncts in regimens aimed at IBD and diseases with similar pathology.

### 6.3 Synergy between polyphenols and probiotics

Probiotics and polyphenols seem to exert a synergistic effect on gut barrier function and mucosal immunity. Probiotic strains like Lactobacillus and Bifidobacterium not only survive better in polyphenol-rich conditions, but are also able to convert the polyphenols into metabolites that exhibit more biological activity and conversely, most of polyphenols and their derivatives can improve the adhesion, stress tolerance and competitive exclusion of pathogens by probiotics (102). Likewise, the combination of probiotics with polyphenol-rich foods or extracts also enhanced the expression of tight junction proteins likes occludin and claudins and reduced intestinal permeability over both alone. All these combinations also facilitated more robust immune modulation, in terms of promoting sIgA generation and maintaining Th17/Treg balance, thus enhancing mucosal protection (103). As a result, fermented functional foods combining specific probiotic strains with customized polyphenol profiles in recent years are considered potential contributors to the targeted manipulation of gut microbiome composition and host immunity (104). These findings support a selective effect of fermentation-derived polyphenol metabolites, particularly in combination with probiotics toward beneficial gut bacteria along with attenuation of inflammatory processes and enhancement of the intestinal barrier function to provide a comprehensive way into gut health.

## 7 Health implications and functional food applications

**Figure 3:**
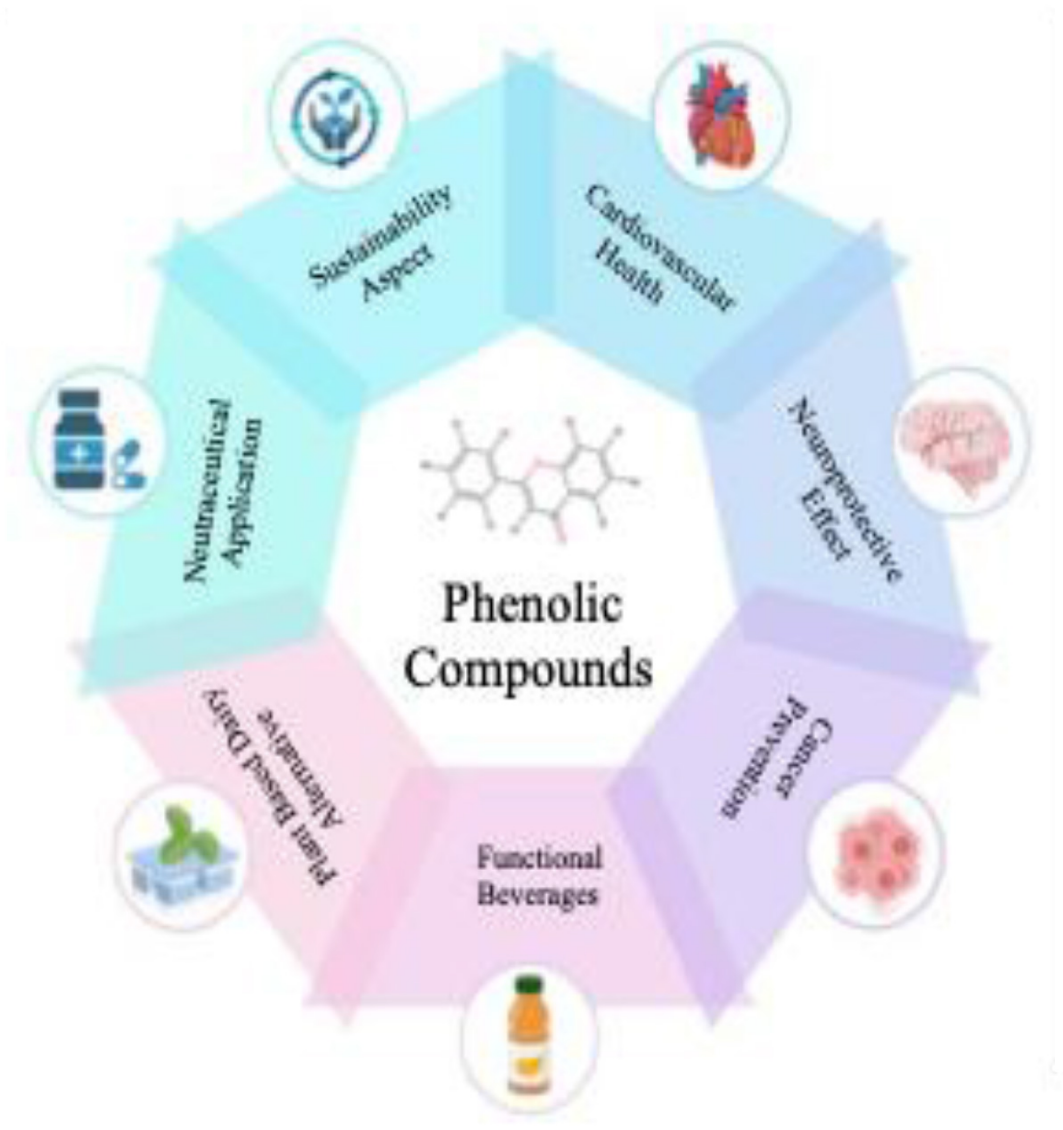
The molecular signalling pathway of polyphenol metabolits.

Polyphenol metabolites, formed during fermentation and subsequent gut microbial metabolism, act not just within the intestine but afar too, affecting cardiovascular, metabolic, neurocognitive and cancer-related pathways systemically (76). Their better bioavailability and structural diversity than dietary natural polyphenols make them to interact with various molecular targets, rendering the fermented polyphenol-rich foods as promising sources for functional food development and disease risk modulation as shown in Figure 3 (105).

### 7.1 Systemic health benefits

#### 7.1.1 Cardiovascular health

Polyphenol consumption showed the improved cardiovascular health while fermentation-based metabolites have been shown to reinforce those associations by improving bioavailability as well as potency (106). Phenolic acid and flavonoid aglycone generated during fermentation process may contribute to the improvement of endothelial function through the upregulation of eNOS expression, enhancement of NO bioavailability, and attenuation of oxidative inactivation of NO, which results in vasodilation and blood pressure regulation. These metabolites also prevent LDL oxidation and downregulate the expression of adhesion molecules and inflammatory markers in vascular endothelium, which is crucial to atherogenesis (107). The consumption of fermented teas, cocoa beverages or polyphenol-enriched fermented dairy and plant-origin drinks demonstrate changes in blood pressure (BP), flow-mediated dilation (FMD) or circulating oxidized low-density lipoprotein (LDL) and C-reactive protein lower than those recorded for non-fermented equivalents thereby increasing the evidence to suggest that fermentation-derived polyphenols help reducing cardiovascular risk markers, what is indeed making an impression on mechanisms of protection against CVD's by fermentative polyphenolic-containing foods in health promotion programs (108).

#### 7.1.2 Neuroprotective effects

**Figure 4:**
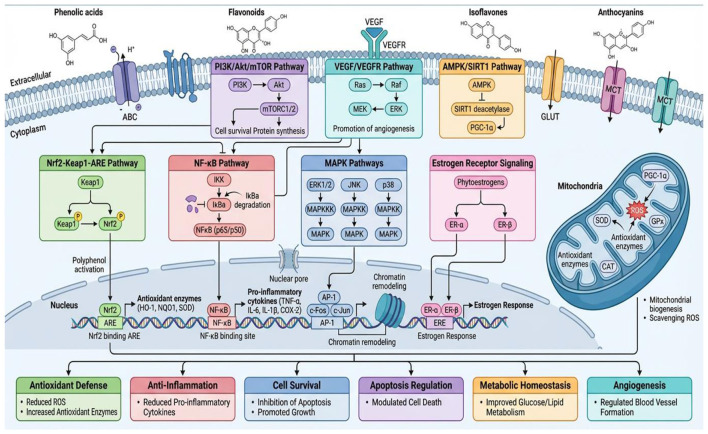
Health implications and functional food applications.

Fermented polyphenol metabolites might exert neuroprotection through direct antioxidative and anti-inflammatory effects in the central nervous system, as well as by modulating the gut-brain axis. Some plogenol acids and flavonoid metabolites representative of microbial metabolism have been shown to be more efficient in their blood-brain barrier penetration with respect to their parent molecules, where they may act directly against ROS, modulate neuronal signaling pathways such as Nrf2, ERK/CREB, and also reduce microglial activation (109). Gut-derived metabolites feed backward from the lumen to shape the composition and metabolic activity of microbiota, resulting in differential production of neuroactive intermediates including short-chain fatty acids (SCFAs), tryptophan derivatives and microbial neurotransmitter analogs signaling directly or indirectly via neural, endocrine and immune pathways with brain impacting effects. Fermented polyphenol-rich diets result in beneficial effects on cognitive performance, reduction of neuroinflammation and attenuation of pathology in models related to neurodegenerative disorders, which are predominantly linked with not only systemic antioxidant status but also favorable alterations in the gut microbiota (110). The molecular signaling pathway of polyphenol metabolits shown in Figure 4.

#### 7.1.3 Prevention of chronic metabolic and neoplastic diseases

Polyphenol metabolites produced through fermentation also are involved in the prevention or reduction of chronic diseases, likes type 2 diabetes, obesity and selected cancers at specific molecular levels. In metabolic control, they can also be beneficial to insulin sensitivity modulating insulin signaling cascades such as PI3K/Akt, increasing glucose uptake and decreasing hepatic gluconeogenesis (111). They may also modulate adipogenesis and energy expenditure by promoting phosphorylation of AMP-activated protein kinase (AMPK) and peroxisome proliferator-activated receptors (PPARs), which in turn limit triacylglycerol accumulation, improve lipid profiles (112). Glycemic management fermented pulse and cereal products enriched with bioactive phenolic acids reported improvements in glycemic control, decrease in postprandial glucose, significant changes on markers of metabolic syndrome when compared with non-fermented controls (113).

In cancer protection, fermentation may lead to higher levels of phenolic metabolites impacting chemical carcinogen metabolism and the modulation of cell proliferation and apoptosis. Some fermentation-derived phenolic acids and flavonoid derivatives cause cell cycle arrest, activate the intrinsic apoptosis pathway, restrict angiogenesis, and obstruct signaling circuits including NF-κB, STAT3, MAPKs in cancer cell lines. On the other hand, fermented pulse polyphenols such as lupins are reported to have a higher anticancer effect *in vitro* than non-fermented extracts as they needed lesser concentration to induce apoptosis and inhibit tumor cell growth, which would be due to the microbial modification of phenolic profile (114). These findings provide support for fermented polyphenol-rich foods as a component of integrated cancer prevention approaches. Different phenolic compound and their health implications are summarized in Table 2.

**Table 2:** Health implications of fermented polyphenol compounds.

**Class**	**Subclass**	**Compounds**	**Metabolites**	**Molecular mechanisms**	**Signaling pathways**	**Health benefits**	**Clinical evidence**	**References**
Phenolic acids	Hydroxycinnamic acids	Ferulic acid, caffeic acid, p-coumaric acid	3-(3-hydroxyphenyl)propionic acid, 3-phenylpropionic acid, benzoic acid derivatives	Nrf2 activation via Keap1 modification; NF-κB p65 nuclear translocation inhibition; enhanced expression of antioxidant enzymes (SOD, CAT, GPx, HO-1)	Nrf2-Keap1-ARE; NF-κB/IκBα/IKK; MAPK (ERK1/2, JNK, p38)	Anti-inflammatory; cardioprotective; neuroprotective; reduced oxidative stress	Reduced inflammatory markers (IL-6, TNF-α, CRP) in clinical trials	(16)
Phenolic acids	Hydroxybenzoic acids	Gallic acid, protocatechuic acid, vanillic acid	Pyrogallol, 4-O-methylgallic acid, hippuric acid	Direct ROS scavenging; metal chelation; inhibition of pro-oxidant enzymes (LOX, COX-2, iNOS)	Nrf2/HO-1; NF-κB inhibition; AP-1 suppression	Anticancer; antimicrobial; cardioprotective; hepatoprotective	Improved endothelial function; reduced blood pressure in hypertensive patients	(124)
Flavonoids	Flavonols	Quercetin, Kaempferol, Rutin	3,4-dihydroxyphenylacetic acid, 3-hydroxyphenylacetic acid, hippuric acid	AMPK activation; PGC-1α upregulation; mitochondrial biogenesis; SIRT1 activation; autophagy induction	AMPK/SIRT1/PGC-1α; PI3K/Akt/mTOR; eNOS/NO/cGMP; FOXO3a	Metabolic health; cardiovascular protection; neuroprotection; longevity promotion	Improved glucose metabolism; enhanced insulin sensitivity; reduced cardiovascular risk markers	(125)
Flavonoids	Flavones	Apigenin, luteolin	Luteolin-7-O-glucuronide, apigenin sulfate conjugates	GABAergic neurotransmission modulation; Anxiety reduction; anti-inflammatory through NF-κB inhibition	GABA-A receptor; NF-κB/COX-2; Nrf2/ARE	Anxiolytic; neuroprotective; anti-inflammatory; anticancer	Reduced anxiety scores in clinical trials; improved sleep quality	(126)
Flavonoids	Flavanones	Naringenin, hesperidin	Naringenin-7-glucuronide, hesperetin conjugates	Lipid metabolism regulation; PPAR-α activation; cholesterol homeostasis	PPAR-α/γ; LXR/RXR; AMPK; SREBP-1c inhibition	Dyslipidemia management; atherosclerosis prevention; metabolic syndrome	Reduced LDL-cholesterol; improved HDL-cholesterol; decreased triglycerides	(127)
Catechins	Flavan-3-ols	EGCG, ECG, EGC, EC	Theabrownins, thearubigins, 5-(3,4-dihydroxyphenyl)-γ-valerolactones	Enhanced bioavailability through microbial transformation; AMPK activation; mitochondrial function improvement; telomerase activity modulation	AMPK/PGC-1α/TFAM; SIRT1/FOXO; PI3K/Akt; eNOS/NO	Vascular function; cognitive enhancement; metabolic health; longevity	PAT index increased 68.9% in high-flavanol group; improved endothelial function	(128)
Anthocyanins	Anthocyanidins	Cyanidin-3-glucoside, delphinidin-3-glucoside, malvidin-3-glucoside	Protocatechuic acid, vanillic acid, syringic acid, gallic acid	BBB permeability enhancement; neuroinflammation reduction; synaptic plasticity support; microglial activation suppression; Aβ aggregation inhibition	Nrf2/HO-1/NQO1; NF-κB/STAT3 suppression; BDNF/TrkB/CREB; PI3K/Akt	Neuroprotection; Alzheimer's/Parkinson's prevention; cardiovascular health; visual function	Improved cognitive function; reduced dementia risk; enhanced memory performance	(129)
Stilbenes	Hydroxystilbenes	Resveratrol, piceatannol, pterostilbene	Dihydroresveratrol, lunularin, resveratrol-3-O-glucuronide	SIRT1 activation; NAD+ boosting; mitochondrial biogenesis; Gut microbiota modulation (↑Bifidobacterium, ↑Prevotella, ↑Lactobacillus); caloric restriction mimetic	SIRT1/PGC-1α/FOXO; AMPK/mTOR; Nrf2/ARE; NF-κB inhibition	Cognitive protection; dementia risk reduction; cardiovascular benefits; longevity; anti-aging	Lower dementia risk with moderate wine consumption; Improved cognitive scores	(130)
Lignans	Mammalian lignans	Enterolactone, Enterodiol (from SDG, Matairesinol)	Enterolactone, enterodiol (gut microbiota-derived)	Estrogen receptor modulation (SERM activity); Antioxidant enzyme induction; Cell cycle arrest (G1 phase); Apoptosis induction in cancer cells	ER-α/ER-β; Nrf2/ARE; NF-κB; p53/Bax/Bcl-2; MAPK	Breast/prostate cancer prevention; Cardiovascular protection; Bone health; Menopausal symptom relief	Reduced breast cancer risk; Improved lipid profiles; Enhanced bone mineral density	(131)
Tannins	Ellagitannins	Punicalagin, Ellagic acid → Urolithins (A, B, C)	Urolithin A, B, C, D (gut microbiota-derived)	Mitophagy induction; mitochondrial quality control; senescent cell clearance; autophagy activation; NAD+ restoration	PINK1/Parkin mitophagy; mTOR; AMPK/ULK1; Nrf2; NF-κB/NLRP3	Anti-aging; muscle health; cognitive function; metabolic health; longevity	Improved muscle strength in elderly; enhanced mitochondrial function; reduced inflammation	(132)
Isoflavones	Soy isoflavones	Daidzein, Genistein, Glycitein → Equol	Equol, O-desmethylangolensin (ODMA), dihydrodaidzein	Enhanced bioavailability (aglycone formation); estrogen receptor modulation; tyrosine kinase inhibition; angiogenesis inhibition; epigenetic modifications	ER-α/ER-β; PI3K/Akt/mTOR; MAPK/ERK; NF-κB; VEGF/VEGFR	Hormone-related cancer prevention; cardiovascular health; Bone density; menopausal relief; metabolic health	Reduced hot flashes; improved bone mineral density; lower cardiovascular disease risk	(125)

### 7.2 Industrial applications as functional foods

There has been growing commercial interest in polyphenol-enriched fermented foods and beverages, plant-based dairy alternatives, and nutraceutical preparations. For example, fermented teas, kombucha-type beverages, and fruit wines have been shown to be rich sources of catechins along with also higher quantities of phenolic acids or other flavonoids which are typically lower in concentration changes during fermentation leading to an increase in antioxidant potential and better sensory properties (115). Likewise, yeast- and bacteria-mediated transformations are employed for enhancing flavor and for evolving the composition of bioactive phenolics and therefore sensory quality associations with functional attributes have been established in fermented cocoa and coffee products (116).

Plant-based dairy substitutes, e.g., fermented soy, oat or almond drinks nowadays be prepared with starter cultures that have been selected for their potential to release and convert inherent or added polyphenols (117). Delivering probiotics with phenolic metabolites by fermentation such as the symbiotic bacteria that produce lactic acid and bifidobacteria can be subjected to co-culture in a fermentation environment with polyphenol-rich substrates in order to generate “synbiotic” beverages that incorporate live bacteria and phenolic metabolites released through the process of bacterial fermentation into one or multiple biologically active compounds targeting well-defined gut, cardiometabolic, and immune health effects (118). Fermentation of plant based drinks preparation or grain based existing beverages with selected microbial consortia not only enhanced the contents of free phenolic acids, antioxidant activities but also improved shelf life, which may facilitate marketing of these functionalized products on a large scale (119).

In addition to beverages, polyphenol-enriched fermented powders, capsules and extracts are being commercialized as a dietary supplement or ingredient for the fortification of bakery, snack and dairy products (120). These compounds are frequently obtained by the fermentation of a fruit, vegetable or pulse matrix with specific strains, drying and standardizing to specific phenolic markers. This strategy permits the boosting of bioactive metabolite levels at more constant doses and their incorporation into a broader range of food formats without potential sensory hurdles linked to high native polyphenol contents, such as astringency or bitterness (121). The industrial application of fermentation to improve polyphenol functionality endorses a well-developing area of the functional food and nutraceutical sectors.

### 7.3 Sustainability aspect of plant-based fermented polyphenols

Fermentation of plant-based polyphenol substrates is also among sustainable food trends as fermentation facilitates production and intake of value-added, food-based products that serve as functional alternatives to animal-based food components and make more efficient use of farming resources. A large number of polyphenol-rich fermentation substrates, such as fruit pomace, cereal bran and legume by-products and other side streams are residues from food processing that would require disposal or be underutilized. Microbial fermentation can valorize such feedstocks to value-added products by releasing phenolics in the bound forms, producing new metabolites and rendering them safe thereby extending shelf life (122). For instance, grape pomace, olive mill waste or cereal bran fermented with LAB, yeasts or molds can lead to powders and extracts rich in bioactive phenolic acids and flavonoids for incorporation into foods, beverages or supplements, thereby reducing any waste generated by the process while uncovering new pathways to revenue.

When compared to many animal-derived functional foods niche likes dairy and meat products, plant fermented polyphenols can potentially involve lower greenhouse gas emissions, land and water footprints especially when based on agroforestry side flows or low input crops. It also targets consumer request for plant-based, “clean label” functional alternatives that meet societal or environmental preferences, or personal dietary needs (123). Through integrating microbiology, food technology and sustainability, fermentation therefore represents an approach to creating functional foods that achieve systemic health effects with a minimum degree of environment burden. Polyphenol-rich fermented foods are now being promoted as potential, possibly sustainable alternatives or supplements to conventional animal-based functional products.

## 8 Challenges and future directions

The investigation of fermented polyphenols is confronted with several challenges, such as the incomplete knowledge on the particular microbial pathways that transform distinct polyphenol structures into bioavailable metabolites. Although several types of enzymes likes β-glucosidases, tannases or decarboxylases have been detected only in poly-fermentations, degradative conversion was typically achieved by a variety of microbial consortia showing redundancy in their capability to convert substrates and contribute toward cooperative or linear degradation processes. This restriction constrains not only mechanistic knowledge but also the rational design of starter cultures toward producing desired polyphenol metabolites. Furthermore, fluctuations in the traditional fermentation process driven by differences in raw material and substrate used, diversity of inocula, factors related to environment and processing also cause inconsistency between the designs and metabolite profiles achieved between different batches. Such variation hinders reproducibility, impedes dose-response knowledge and may allow the production of off-flavors or contaminants, underlining the necessity for controlled and standardized fermentations. Furthermore, there is a lack of effective translation from good *in vitro* and animal experimental evidence to strong human clinical data. Current human studies are studying products that are often of variable batch and content, not well phenotyped for their polyphenol profile and experiencing great per individual differences in microbiota, genetics and life-style which determine responsiveness. In the absence of comprehensive omics data spanning these trials, it is difficult to definitively connect specific fermentation-derived polyphenol metabolites to clinical effects and illustrates how urgently require controlled multi-arm human studies with accurate compositional and functional readouts.

Further developments will depend on precision fermentation, which involves using specific microbial communities, standardized substrates and controlled conditions that allow for the repeated synthesis of selected polyphenol metabolites. Through careful selection of strains with defined enzymatic profile and fine-tuning pH, temperature, nutrient concentration, specific transformations likes isoflavone deglycosylation, hydroxy-cinnamic acid de-esterification can be promoted over non-desirable side products. Adaptive control of fermentation by analytical technologies for real-time monitoring and specification-based corrective action can move from empirical to predictable “fermentation”. Synthetic biology offers a suite of advanced tools to tailor-make microbes with either enhanced or unprecedented polyphenol-transforming pathways that are more efficient and reliable at producing particular bioactive metabolites. However, regulatory, safety and acceptance among consumers poses careful consideration before use of engineered strains will be widely accepted.

Broadening multiomics systems including integrated metagenomics, metatranscriptomics, proteomics, and metabolomics applications is transforming the capabilities to understand fermentation systems through community composition, gene expression, enzyme activity, and metabolite dynamics connections. Time-resolved multi-omics at high resolution allows for predictive mechanistic modeling and rational optimization of fermentations by providing insights into how the microbial ecology and metabolic pathways respond to variation in strain choice, substrate composition, or environmental conditions. Integrating multi-omics into clinical nutrition also offers potential advances in precision diets through linking fermented food metabolite profiles to the changes in gut microbiome, host metabolic responses, and health status. Although real-time monitoring and adaptive interventions are technically challenging, these constitute the frontier for personalized nutrition with fermented polyphenols. And lastly, the incorporation of safety evaluations and sustainability concepts is critical. Contaminant profiling for mycotoxins and biogenic amines helps guarantee that fermented products are safe for broad consumption. By the same token, the valorization of agri-food by-products and side streams from plants for polyphenol fermentation is in line with a circular economy approach minimizing environmental footprints compared to animal-based functional foods. Multi-criteria assessments including nutrition, health, safety and environmental impact will be used to guide the design of effective, safe and sustainable fermented polyphenol-based products. In conclusion, the current bottleneck of mechanistic understanding, standardization and clinical validation can be tackled by precision fermentation, synthetic biology and by comprehensive multi-omics to reveal the full potential of fermented polyphenols. This integrated pipeline promotes the exploration of new generation functional foods and personalized nutrition interventions for better public health.

## 9 Conclusion

Polyphenols, bioactive compounds in plant-based foods, are known for their antioxidant, anti-inflammatory, and antimicrobial properties, but their bioactivity and bioavailability are limited by their complex structures. Fermentation enhances polyphenols' bioavailability and bioactivity by converting them into more bioavailable metabolites with improved solubility, stability, and antioxidant properties. These transformations increase their absorption and health benefits. Fermented polyphenol metabolites also modulate gut microbiota, promoting beneficial bacteria like *Lactobacillus* and *Bifidobacterium*, supporting gut health, reducing inflammation, and offering systemic benefits, including improved metabolic, immune, and neurocognitive health. However, gaps remain in understanding the microbial pathways of polyphenol transformation and their health outcomes. Future research should focus on detailed metabolite analysis, multi-omics approaches, and human clinical trials to validate these health benefits. Advancements in fermentation technology, including precision fermentation, can further optimize polyphenol-enriched functional foods, utilizing underutilized food by-products and offering sustainable solutions for both health and environmental challenges.

## References

[1] Rudrapal M Khairnar SJ Khan J Bin DA Ansari MA Alomary MN . Dietary polyphenols and their role in oxidative stress-induced human diseases: insights into protective effects, antioxidant potentials and mechanism(s) of action. Front Pharmacol. (2022) 13:806470. doi: 10.3389/fphar.2022.806470 35237163 PMC8882865

[2] Bravo L. Polyphenols: chemistry, dietary sources, metabolism, and nutritional significance. Nutr Rev. (1998) 56:317–33. doi: 10.1111/j.1753-4887.1998tb01670.x 9838798

[3] Zhang Z Li X Sang S McClements DJ Chen L Long J . Polyphenols as plant-based nutraceuticals: health effects, encapsulation, nano-delivery, and application. Foods. (2022) 11:2189. doi: 10.3390/foods11152189 35892774 PMC9330871

[4] Bié J Sepodes B Fernandes PCB Ribeiro MHL. Polyphenols in health and disease: gut microbiota, bioaccessibility, and bioavailability. Compounds. (2023) 3:40–72. doi: 10.3390/compounds3010005

[5] Yahfoufi N Alsadi N Jambi M Matar C. The immunomodulatory and anti-inflammatory role of polyphenols. Nutrients. (2018) 10:1618. doi: 10.3390/nu10111618 30400131 PMC6266803

[6] Mahdi L Graziani A Baffy G Mitten EK Portincasa P Khalil M. Unlocking polyphenol efficacy : the role of gut microbiota in modulating bioavailability and health effects. Nutrients. (2025) 17:1–35. doi: 10.3390/nu17172793 PMC12430036 40944183

[7] Marco ML Heeney D Binda S Cifelli CJ Cotter PD Foligné B . Health benefits of fermented foods: microbiota and beyond. Curr Opin Biotechnol. (2017) 44:94–102. doi: 10.1016/j.copbio.2016.11.010 27998788

[8] Tamang JP Watanabe K Holzapfel WH. Review: diversity of microorganisms in global fermented foods and beverages. Front Microbiol. (2016) 7:377. doi: 10.3389/fmicb.2016.00377 27047484 PMC4805592

[9] Park I Mannaa M. Fermented foods as functional systems: microbial communities and metabolites influencing gut health and systemic outcomes. Foods. (2025) 14:1–15. doi: 10.3390/foods14132292 40647044 PMC12249102

[10] Fitsum S Gebreyohannes G Sbhatu DB. Bioactive compounds in fermented foods: health benefits, safety, and future perspectives. Appl Food Res. (2025) 5:101097. doi: 10.1016/j.afres.2025.101097

[11] Yang F Chen C Ni D Yang Y Tian J Li Y . Effects of fermentation on bioactivity and the composition of polyphenols contained in polyphenol-rich foods: a review. Foods. (2023) 12:3315. doi: 10.3390/foods12173315 37685247 PMC10486714

[12] Leonard W Zhang P Ying D Adhikari B Fang Z. Fermentation transforms the phenolic profiles and bioactivities of plant-based foods. Biotechnol Adv. (2021) 49:107763. doi: 10.1016/j.biotechadv.2021.107763 33961978

[13] Luca SV Macovei I Bujor A Miron A Skalicka-Wozniak K Aprotosoaie AC . Bioactivity of dietary polyphenols: the role of metabolites. Crit Rev Food Sci Nutr. (2020) 60:626–59. doi: 10.1080/10408398.2018.1546669 30614249

[14] Hu J Mesnage R Tuohy K Heiss C Rodriguez-Mateos A. (Poly)phenol-related gut metabotypes and human health: an update. Food Funct. (2024) 15:2814–35. doi: 10.1039/D3FO04338J 38414364

[15] Zhang B Zhang Y Xing X Wang S. Health benefits of dietary polyphenols: insight into interindividual variability in absorption and metabolism. Curr Opin Food Sci. (2022) 48:100941. doi: 10.1016/j.cofs.2022.100941

[16] Kumar A Saranyadevi S Thirumalaisamy SK Dapana Durage TT Jaiswal SG Kavitake D . Phenolic acids in fermented foods: microbial biotransformation, antioxidant mechanisms, and functional health implications. Front Mol Biosci. (2025) 12:1678673. doi: 10.3389/fmolb.2025.1678673 41195418 PMC12583000

[17] Wang X Qi Y Zheng H. Dietary polyphenol, gut microbiota, and health benefits. Antioxidants. (2022) 11:1212. doi: 10.3390/antiox11061212 35740109 PMC9220293

[18] Rajha HN Paule A Aragonès G Barbosa M Caddeo C Debs E . Recent advances in research on polyphenols: effects on microbiota, metabolism, and health. Mol Nutr Food Res. (2022) 66:e2100670. doi: 10.1002/mnfr.202100670 34806294

[19] Manach C Scalbert A Morand C Rémésy C Jiménez L. Polyphenols: food sources and bioavailability. Am J Clin Nutr. (2004) 79:727–47. doi: 10.1093/ajcn/79.5.727 15113710

[20] Tsao R. Chemistry and biochemistry of dietary polyphenols. Nutrients. (2010) 2:1231–46. doi: 10.3390/nu2121231 22254006 PMC3257627

[21] Hegde MM Lakshman K. Role of polyphenols and flavonoids as anti-cancer drug candidates: a review. Pharmacognosy Res. (2023) 15:206–16. doi: 10.5530/pres.15.2.022

[22] Siemińska-Kuczer A Szymańska-Chargot M Zdunek A. Recent advances in interactions between polyphenols and plant cell wall polysaccharides as studied using an adsorption technique. Food Chem. (2022) 373:131487. doi: 10.1016/j.foodchem.2021.131487 34741970

[23] Williamson G. Effects of polyphenols on glucose-induced metabolic changes in healthy human subjects and on glucose transporters. Mol Nutr Food Res. (2022) 66:e2101113. doi: 10.1002/mnfr.202101113 35315210 PMC9788283

[24] Lang Y Gao N Zang Z Meng X Lin Y Yang S . Classification and antioxidant assays of polyphenols: a review. J Future Foods. (2024) 4:193–204. doi: 10.1016/j.jfutfo.2023.07.002

[25] Aatif M. Current understanding of polyphenols to enhance bioavailability for better therapies. Biomedicines. (2023) 11:2078. doi: 10.3390/biomedicines11072078 37509717 PMC10377558

[26] Padilla-González GF Grosskopf E Sadgrove NJ Simmonds MSJ. Chemical diversity of Flavan-3-Ols in grape seeds: modulating factors and quality requirements. Plants. (2022) 11:809. doi: 10.3390/plants11060809 35336690 PMC8953305

[27] Jang CH Oh J Lim JS Kim HJ Kim JS. Fermented soy products: beneficial potential in neurodegenerative diseases. Foods. (2021) 10:636. doi: 10.3390/foods10030636 33803607 PMC8003083

[28] Debelo H Li M Ferruzzi MG. Processing influences on food polyphenol profiles and biological activity. Curr Opin Food Sci. (2020) 32:90–102. doi: 10.1016/j.cofs.2020.03.001

[29] Khan MK Ahmad K Hassan S Imran M Ahmad N Xu C. Effect of novel technologies on polyphenols during food processing. Innov Food Sci Emerg Technol. (2018) 45:361–81. doi: 10.1016/j.ifset.2017.12.006

[30] Gaur G Gänzle MG. Conversion of (poly)phenolic compounds in food fermentations by lactic acid bacteria: novel insights into metabolic pathways and functional metabolites. Curr Res Food Sci. (2023) 6:100448. doi: 10.1016/j.crfs.2023.100448 36713641 PMC9876838

[31] Zhang H Yu H. Enhanced biotransformation of soybean isoflavone from glycosides to aglycones using solid-state fermentation of soybean with effective microorganisms (EM) strains. J Food Biochem. (2019) 43:e12804. doi: 10.1111/jfbc.12804 31353590

[32] Sarkar P Abedin MM Singh SP Pandey A Rai AK. Microbial production and transformation of polyphenols. Curr Dev Biotechnol Bioeng. (2021) 2022:189–208. doi: 10.1016/B978-0-12-823506-5.00005-9

[33] Adebo OA Medina-Meza IG. Impact of fermentation on the phenolic compounds and antioxidant activity of whole cereal grains: a mini review. Molecules. (2020) 25:927. doi: 10.3390/molecules25040927 32093014 PMC7070691

[34] Nyhan LM Lynch KM Sahin AW Arendt EK. Advances in Kombucha tea fermentation: a review. Appl Microbiol. (2022) 2:73–103. doi: 10.3390/applmicrobiol2010005

[35] Di Pede G Mena P Bresciani L Achour M Lamuela-Raventós RM Estruch R . Revisiting the bioavailability of flavan-3-ols in humans: a systematic review and comprehensive data analysis. Mol Aspects Med. (2023) 89:101146. doi: 10.1016/j.mam.2022.101146 36207170

[36] Schroeter H Heiss C Balzer J Kleinbongard P Keen CL Hollenberg NK . (-)-Epicatechin mediates beneficial effects of flavanol-rich cocoa on vascular function in humans. Proc Natl Acad Sci U S A. (2006) 103:1024–9. doi: 10.1073/pnas.0510168103 16418281 PMC1327732

[37] Bladé C Aragonès G Arola-Arnal A Muguerza B Bravo FI Salvadó MJ . Proanthocyanidins in health and disease. BioFactors. (2016) 42:5–12. doi: 10.1002/biof.1249 26762288

[38] Khoo HE Azlan A Tang ST Lim SM. Anthocyanidins and anthocyanins: colored pigments as food, pharmaceutical ingredients, and the potential health benefits. Food Nutr Res. (2017) 61:1361779. doi: 10.1080/16546628.2017.1361779 28970777 PMC5613902

[39] Lila MA Burton-Freeman B Grace M Kalt W. Unraveling anthocyanin bioavailability for human health. Annu Rev Food Sci Technol. (2016) 7:375–93. doi: 10.1146/annurev-food-041715-033346 26772410

[40] Fernandes I Faria A Calhau C de Freitas V Mateus N. Bioavailability of anthocyanins and derivatives. J Funct Foods. (2014) 7:54–66. doi: 10.1016/j.jff.2013.05.010

[41] Cassidy A. Berry anthocyanin intake and cardiovascular health. Mol Aspects Med. (2018) 61:76–82. doi: 10.1016/j.mam.2017.05.002 28483533

[42] Mlcek J Jurikova T Skrovankova S Sochor J. Quercetin and its anti-allergic immune response. Molecules. (2016) 21:623. doi: 10.3390/molecules21050623 27187333 PMC6273625

[43] M. Calderon-Montano J, Burgos-Moron E, Perez-Guerrero C, Lopez-Lazaro M. A review on the dietary flavonoid kaempferol. Mini Rev Med Chem. (2011) 11:298–344. doi: 10.2174/138955711795305335 21428901

[44] Chen AY Chen YC. A review of the dietary flavonoid, kaempferol on human health and cancer chemoprevention. Food Chem. (2013) 138:2099–10. doi: 10.1016/j.foodchem.2012.11.139 23497863 PMC3601579

[45] Semwal DK Semwal RB Combrinck S Viljoen A. Myricetin: a dietary molecule with diverse biological activities. Nutrients. (2016) 8:90. doi: 10.3390/nu8020090 26891321 PMC4772053

[46] Monteiro Espíndola KM Ferreira RG Mosquera Narvaez LE Rocha Silva Rosario AC Machado Da Silva AH Bispo Silva AG . Chemical and pharmacological aspects of caffeic acid and its activity in hepatocarcinoma. Front Oncol. (2019) 9:541. doi: 10.3389/fonc.2019.00541 31293975 PMC6598430

[47] Zduńska K Dana A Kolodziejczak A Rotsztejn H. Antioxidant properties of ferulic acid and its possible application. Skin Pharmacol Physiol. (2018) 31:332–6. doi: 10.1159/000491755 30235459

[48] Boz H. p-coumaric acid in cereals: presence, antioxidant and antimicrobial effects. Int J Food Sci Technol. (2015) 50:2323–28. doi: 10.1111/ijfs.12898

[49] Baur JA Sinclair DA. Therapeutic potential of resveratrol: the *in vivo* evidence. Nat Rev Drug Discov. (2006) 5:493–506. doi: 10.1038/nrd2060 16732220

[50] Kahkeshani N Farzaei F Fotouhi M Alavi SS Bahramsoltani R Naseri R . Pharmacological effects of gallic acid in health and disease: a mechanistic review. Iran J Basic Med Sci. (2019) 22:225–37. doi: 10.22038/ijbms.2019.32806.7897 31156781 PMC6528712

[51] Kakkar S Bais S. A review on protocatechuic acid and its pharmacological potential. ISRN Pharmacol. (2014) 2014:952943. doi: 10.1155/2014/952943 25006494 PMC4005030

[52] Setchell KDR Clerici C. Equol: history, chemistry, and formation. J Nutr. (2010) 140:1355S−62S. doi: 10.3945/jn.109.119776 20519412 PMC2884333

[53] Spagnuolo C Russo GL Orhan IE Habtemariam S Daglia M Sureda A . Genistein and cancer: current status, challenges, and future directions. Adv Nutr. (2015) 6:408–19. doi: 10.3945/an.114.008052 26178025 PMC4496735

[54] Zaheer K Humayoun Akhtar M. An updated review of dietary isoflavones: nutrition, processing, bioavailability and impacts on human health. Crit Rev Food Sci Nutr. (2017) 57:1280–93. doi: 10.1080/10408398.2014.989958 26565435

[55] Rauf A Imran M Abu-Izneid T. Iahtisham-Ul-Haq, Patel S, Pan X, et al. Proanthocyanidins: a comprehensive review. Biomed Pharmacother. (2019) 116:108999. doi: 10.1016/j.biopha.2019.108999 31146109

[56] Leung LK Su Y Chen R Zhang Z Huang Y Chen ZY. Theaflavins in black tea and catechins in green tea are equally effective antioxidants. J Nutr. (2001) 131:2248–51. doi: 10.1093/jn/131.9.2248 11533262

[57] Lv H Zhang Y Lin Z Liang Y. Processing and chemical constituents of Pu-erh tea: a review. Food Res Int. (2013) 53:608–18. doi: 10.1016/j.foodres.2013.02.043

[58] Sergides C Chirilă M Silvestro L Pitta D Pittas A. Bioavailability and safety study of resveratrol 500 mg tablets in healthy male and female volunteers. Exp Ther Med. (2016) 11:164–70. doi: 10.3892/etm.2015.2895 26889234 PMC4726856

[59] Remsberg CM Yáñez JA Ohgami Y Vega-Villa KR Rimando AM Davies NM. Pharmacometrics of pterostilbene: preclinical pharmacokinetics and metabolism, anticancer, antiinflammatory, antioxidant and analgesic activity. Phytother Res. (2008) 22:169–79. doi: 10.1002/ptr.2277 17726731

[60] Espín JC Larrosa M García-Conesa MT Tomás-Barberán F. Biological significance of urolithins, the gut microbial ellagic acid-derived metabolites: the evidence so far. Evid Based Complement Alternat Med. (2013) 2013:270418. doi: 10.1155/2013/270418 23781257 PMC3679724

[61] Peterson J Dwyer J Adlercreutz H Scalbert A Jacques P McCullough ML. Dietary lignans: physiology and potential for cardiovascular disease risk reduction. Nutr Rev. (2010) 68:571–603. doi: 10.1111/j.1753-4887.2010.00319.x 20883417 PMC2951311

[62] Tajik N Tajik M Mack I Enck P. The potential effects of chlorogenic acid, the main phenolic components in coffee, on health: a comprehensive review of the literature. Eur J Nutr. (2017) 56:2215–44. doi: 10.1007/s00394-017-1379-1 28391515

[63] Parhiz H Roohbakhsh A Soltani F Rezaee R Iranshahi M. Antioxidant and anti-inflammatory properties of the citrus flavonoids hesperidin and hesperetin: an updated review of their molecular mechanisms and experimental models. Phytother Res. (2015) 29:323–31. doi: 10.1002/ptr.5256 25394264

[64] Nehlig A. Effects of coffee/caffeine on brain health and disease: what should i tell my patients? Pract Neurol. (2016) 16:89–95. doi: 10.1136/practneurol-2015-001162 26677204

[65] Martínez-Pinilla E Oñatibia-Astibia A Franco R. The relevance of theobromine for the beneficial effects of cocoa consumption. Front Pharmacol. (2015) 6:30. doi: 10.3389/fphar.2015.00030 25750625 PMC4335269

[66] Saad AM Mohammed DM Alkafaas SS Ghosh S Negm SH Salem HM . Dietary polyphenols and human health: sources, biological activities, nutritional and immunological aspects, and bioavailability– a comprehensive review. Front Immunol. (2025) 16:1–44. doi: 10.3389/fimmu.2025.1653378 41256860 PMC12620276

[67] Polia F Pastor-Belda M Martínez-Blázquez A Horcajada MN Tomás-Barberán FA García-Villalba R. Technological and biotechnological processes to enhance the bioavailability of dietary (Poly)phenols in humans. J Agric Food Chem. (2022) 70:2092–107. doi: 10.1021/acs.jafc.1c07198 35156799 PMC8880379

[68] Hur SJ Lee SY Kim YC Choi I Kim GB. Effect of fermentation on the antioxidant activity in plant-based foods. Food Chem. (2014) 160:346–56. doi: 10.1016/j.foodchem.2014.03.112 24799248

[69] Yousefi N Shokrollahi Yancheshmeh B Gernaey K V. The potential of fermentation-based processing on protein modification: a review. Foods. (2025) 14:3461. doi: 10.3390/foods14203461 41153997 PMC12562408

[70] Sejbuk M Mirończuk-Chodakowska I Karav S Witkowska AM. Dietary polyphenols, food processing and gut microbiome: recent findings on bioavailability, bioactivity, and gut microbiome interplay. Antioxidants. (2024) 13:1220. doi: 10.3390/antiox13101220 39456473 PMC11505337

[71] Tušek K Valinger D Jurina T Sokač Cvetnić T Gajdoš Kljusurić J Benković M. Bioactives in cocoa: novel findings, health benefits, and extraction techniques. Separations. (2024) 11:128. doi: 10.3390/separations11040128

[72] Teng H Chen L. Polyphenols and bioavailability: an update. Crit Rev Food Sci Nutr. (2019) 59:2040–51. doi: 10.1080/10408398.2018.1437023 29405736

[73] Xie F Yang W Xing M Zhang H Ai L. Natural polyphenols-gut microbiota interactions and effects on glycolipid metabolism via polyphenols-gut-brain axis: a state-of-the-art review. Trends Food Sci Technol. (2023) 140:104171. doi: 10.1016/j.tifs.2023.104171

[74] do Prado FG Pagnoncelli MGB de Melo Pereira GV Karp SG Soccol CR. Fermented soy products and their potential health benefits: a review. Microorganisms. (2022) 10:1606. doi: 10.3390/microorganisms10081606 36014024 PMC9416513

[75] Singaravelan N Tollefsbol TO. Polyphenol-based prevention and treatment of cancer through epigenetic and combinatorial mechanisms. Nutrients. (2025) 17:616. doi: 10.3390/nu17040616 40004944 PMC11858336

[76] Kilua A Nagata R Han KH Fukushima M. Beneficial health effects of polyphenols metabolized by fermentation. Food Sci Biotechnol. (2022) 31:1027–40. doi: 10.1007/s10068-022-01112-0 35873377 PMC9300792

[77] Nemzer B V Al-Taher F Kalita D Yashin AY Yashin YI. Health-improving effects of polyphenols on the human intestinal microbiota: a review. Int J Mol Sci. (2025) 26:1–20. doi: 10.3390/ijms26031335 39941107 PMC11818678

[78] Rodríguez-Daza MC Pulido-Mateos EC Lupien-Meilleur J Guyonnet D Desjardins Y Roy D. Polyphenol-mediated gut microbiota modulation: toward prebiotics and further. Front Nutr. (2021) 8:689456. doi: 10.3389/fnut.2021.689456 34268328 PMC8276758

[79] Sun C Zhao C Guven EC Paoli P Simal-Gandara J Ramkumar KM . Dietary polyphenols as antidiabetic agents: advances and opportunities. Food Front. (2020) 1. doi: 10.1002/fft2.15

[80] Iglesias-Aguirre CE Cortés-Martín A Ávila-Gálvez MA Giménez-Bastida JA Selma MV González-Sarrías A . Main drivers of (poly)phenol effects on human health: metabolite production and/or gut microbiota-associated metabotypes? Food Funct. (2021) 12:10324–55. doi: 10.1039/D1FO02033A 34558584

[81] Sawant SS Park HY Sim EY Kim HS Choi HS. Microbial fermentation in food: impact on functional properties and nutritional enhancement—a review of recent developments. Fermentation. (2025) 11:15. doi: 10.3390/fermentation11010015

[82] Molina GES Ras G da Silva DF Duedahl-Olesen L Hansen EB Bang-Berthelsen CH. Metabolic insights of lactic acid bacteria in reducing off-flavors and antinutrients in plant-based fermented dairy alternatives. Compr Rev Food Sci Food Saf. (2025) 24:1–34. doi: 10.1111/1541-4337.70134 40091739 PMC11911983

[83] Paventi G Di Martino C Coppola F Iorizzo M. β-glucosidase activity of lactiplantibacillus plantarum: a key player in food fermentation and human health. Foods. (2025) 14:1–28. doi: 10.20944/preprints202503.1795.v1 PMC12072041 40361534

[84] Liu M Fang Y Chen R Cai M Yang X Fang Z . Effect of lactobacillus plantarum 1243 fermentation on quality properties and metabolome of Aronia melanocarpa (Michx.) elliott juice. Food Chem X. (2025) 29:102706. doi: 10.1016/j.fochx.2025.102706 40672877 PMC12266521

[85] Liu M Zhang L Li J Xu G Zong W Wang L. Effects of lactic acid bacteria on antioxidant activity *in vitro* and aroma component of Eucommia ulmoides tea. J Food Sci Technol. (2024) 61:169–77. doi: 10.1007/s13197-023-05833-w 38192710 PMC10771573

[86] Yuan X Wang T Sun L Qiao Z Pan H Zhong Y . Recent advances of fermented fruits: a review on strains, fermentation strategies, and functional activities. Food Chem X. (2024) 22:101482. doi: 10.1016/j.fochx.2024.101482 38817978 PMC11137363

[87] Gutiérrez-Ríos HG Suárez-Quiroz ML Hernández-Estrada ZJ Castellanos-Onorio OP Alonso-Villegas R Rayas-Duarte P . Yeasts as producers of flavor precursors during cocoa bean fermentation and their relevance as starter cultures: a review. Fermentation. (2022) 8:331. doi: 10.3390/fermentation8070331

[88] Chen W Lv X Tran VT Maruyama JI Han KH Yu JH. Editorial: from traditional to modern: progress of molds and yeasts in fermented-food production. Front Microbiol. (2022) 13:876872. doi: 10.3389/fmicb.2022.876872 35401444 PMC8992652

[89] Sun X Jiang M Lan Y Duan C Yan G. Co-fermentation of L. plantarum and enological yeasts enhances the quality of a multi-substrate fermented beverage: physicochemical, bioactive and flavor profiles. Food Biosci. (2025) 69:106942. doi: 10.1016/j.fbio.2025.106942

[90] Cryan JF. O'riordan KJ, Cowan CSM, Sandhu KV, Bastiaanssen TFS, Boehme M, et al. The microbiota-gut-brain axis. Physiol Rev. (2019) 99:1877–2013. doi: 10.1152/physrev.00018.2018 31460832

[91] Li Y He W Liu S Hu X He Y Song X . Innovative omics strategies in fermented fruits and vegetables: unveiling nutritional profiles, microbial diversity, and future prospects. Compr Rev Food Sci Food Saf. (2024) 23:e70030. doi: 10.1111/1541-4337.70030 39379298

[92] Ji J Jiang X Song P Yang Q Sun M Dong Z . Multi-omics insights into microbial interactions and fermented food quality. Microorganisms. (2025) 13:2679. doi: 10.3390/microorganisms13122679 41471883 PMC12734765

[93] Xie M Wang J Wang F Wang J Yan Y Feng K . A review of genomic, transcriptomic, and proteomic applications in edible fungi biology: current status and future directions. J Fungi. (2025) 11:422. doi: 10.3390/jof11060422 40558935 PMC12194055

[94] Chuzel L Fossa SL Boisvert ML Cajic S Hennig R Ganatra MB . Combining functional metagenomics and glycoanalytics to identify enzymes that facilitate structural characterization of sulfated N-glycans. Microb Cell Fact. (2021) 20:162. doi: 10.1186/s12934-021-01652-w 34419057 PMC8379841

[95] Tiozon RJN Sartagoda KJD Serrano LMN Fernie AR Sreenivasulu N. Metabolomics based inferences to unravel phenolic compound diversity in cereals and its implications for human gut health. Trends Food Sci Technol. (2022) 127:14–25. doi: 10.1016/j.tifs.2022.06.011 36090468 PMC9449372

[96] Ciupei D Colişar A Leopold L Stănilă A Diaconeasa ZM. Polyphenols: from classification to therapeutic potential and bioavailability. Foods. (2024) 13:4131. doi: 10.3390/foods13244131 39767073 PMC11675957

[97] Mithul Aravind S Wichienchot S Tsao R Ramakrishnan S Chakkaravarthi S. Role of dietary polyphenols on gut microbiota, their metabolites and health benefits. Food Res Int. (2021) 142:110189. doi: 10.1016/j.foodres.2021.110189 33773665

[98] Costa EM Silva S. Impact of polyphenols on human gut microbiome and associated biomarkers. In: Technologies to Recover Polyphenols from AgroFood By-products and Wastes. Amsterdam: Elsevier (2022). p. 25–40. doi: 10.1016/B978-0-323-85273-9.00005-3

[99] Zhao Y Jiang Q. Roles of the polyphenol-gut microbiota interaction in alleviating colitis and preventing colitis-associated colorectal cancer. Adv Nutr. (2021) 12:546–65. doi: 10.1093/advances/nmaa104 32905583 PMC8009754

[100] Fraga CG Croft KD Kennedy DO Tomás-Barberán FA. The effects of polyphenols and other bioactives on human health. Food Funct. (2019) 10:514–28. doi: 10.1039/C8FO01997E 30746536

[101] Piekarska-Radzik L Klewicka E. Mutual influence of polyphenols and Lactobacillus spp. bacteria in food: a review. Eur Food Res Technol. (2021) 247:9–24. doi: 10.1007/s00217-020-03603-y

[102] Taverniti V Del Bo' C Fiore W Gargari G Arioli S Riso P . Combination of different probiotics and berry-derived (poly)phenols can modulate immune response in dendritic cells. J Funct Foods. (2022) 94:105121. doi: 10.1016/j.jff.2022.105121

[103] Wang K Hu S. The synergistic effects of polyphenols and intestinal microbiota on osteoporosis. Front Immunol. (2023) 14:1285621. doi: 10.3389/fimmu.2023.1285621 37936705 PMC10626506

[104] Valentino V Magliulo R Farsi D Cotter PD O'Sullivan O Ercolini D . Fermented foods, their microbiome and its potential in boosting human health. Microb Biotechnol. (2024) 17:e14428. doi: 10.1111/1751-7915.14428 38393607 PMC10886436

[105] Silva RFM Pogačnik L. Food, polyphenols and neuroprotection. Neural Regen Res. (2017) 12:582–3. doi: 10.4103/1673-5374.205096 28553336 PMC5436354

[106] Iqbal I Wilairatana P Saqib F Nasir B Wahid M Latif MF . Plant polyphenols and their potential benefits on cardiovascular health: a review. Molecules. (2023) 28:6403. doi: 10.3390/molecules28176403 37687232 PMC10490098

[107] Bianchi F Cappella A Gagliano N Sfondrini L Stacchiotti A. Polyphenols–gut–heart: an impactful relationship to improve cardiovascular diseases. Antioxidants. (2022) 11:1700. doi: 10.3390/antiox11091700 36139775 PMC9495581

[108] Jalil AMM Ismail A. Polyphenols in cocoa and cocoa products: is there a link between antioxidant properties and health? Molecules. (2008) 13:2190–219. doi: 10.3390/molecules13092190 18830150 PMC6245372

[109] Nazzaro F Coppola F Fratianni F Abdalrazeq M Ombra MN De Giulio B . Polyphenols bioactive metabolites, and their anti-biofilm and neuroprotective potential. Foods. (2025) 14:3976. doi: 10.3390/foods14223976 41300132 PMC12651691

[110] Zhang W Dong X Huang R. Antiparkinsonian effects of polyphenols: a narrative review with a focus on the modulation of the gut-brain axis. Pharmacol Res. (2023) 193:106787. doi: 10.1016/j.phrs.2023.106787 37224894

[111] de Paulo Farias D de Araújo FF Neri-Numa IA Pastore GM. Antidiabetic potential of dietary polyphenols: a mechanistic review. Food Res Int. (2021) 145:110383. doi: 10.1016/j.foodres.2021.110383 34112386

[112] Song H Jia W. Beneficial effects of food-derived polyphenols on type 2 diabetes: mechanistic insights based on gut microbiota alterations and anti-inflammatory responses. Food Sci Anim Products. (2023) 1:9240043. doi: 10.26599/FSAP.2023.9240043

[113] Aloo SO Ofosu FK Kim NH Kilonzi SM Oh DH. Insights on dietary polyphenols as agents against metabolic disorders: obesity as a target disease. Antioxidants. (2023) 12:416. doi: 10.3390/antiox12020416 36829976 PMC9952395

[114] Bakrim S El Omari N El Hachlafi N Bakri Y Lee LH Bouyahya A. Dietary phenolic compounds as anticancer natural drugs: recent update on molecular mechanisms and clinical trials. Foods. (2022) 11:3323. doi: 10.3390/foods11213323 36359936 PMC9657352

[115] Arshad Z Shahid S Hasnain A Yaseen E Rahimi M. Functional foods enriched with bioactive compounds: therapeutic potential and technological innovations. Food Sci Nutr. (2025) 13:e71024. doi: 10.1002/fsn3.71024 41063746 PMC12501769

[116] Doriya K Kumar DS Thorat BN. A systematic review on fruit-based fermented foods as an approach to improve dietary diversity. J Food Process Preserv. (2022) 46. doi: 10.1111/jfpp.16994

[117] Harper AR Dobson RCJ Morris VK Moggré GJ. Fermentation of plant-based dairy alternatives by lactic acid bacteria. Microb Biotechnol. (2022) 15:1404–21. doi: 10.1111/1751-7915.14008 35393728 PMC9049613

[118] Huang W Wätjen AP Prakash S Bang-Berthelsen CH Turner MS. Exploring lactic acid bacteria diversity for better fermentation of plant-based dairy alternatives. Microbiol Aust. (2022) 43:79–82. doi: 10.1071/MA22026

[119] Viretto C Tlais AZA Tuccillo F Polo A Arora K Verté F . Maximize the synergistic interactions among microbial consortia and plant-based matrices to design fermented cereal-pulse-based beverages. Food Res Int. (2025) 220:117045. doi: 10.1016/j.foodres.2025.117045 41074290

[120] Chernenko S. Encapsulation of polyphenols in baked goods: a strategy for enhancing stability and antioxidant activity. Technol Audit Prod Reserves. (2025) 4:45–51. doi: 10.15587/2706-5448.2025.332998

[121] Ronie ME Abdul Aziz AH Kobun R Pindi W Roslan J Putra NR . Unveiling the potential applications of plant by-products in food – a review. Waste Manag Bull. (2024) 2:183–203. doi: 10.1016/j.wmb.2024.07.008

[122] de Oliveira I Santos-Buelga C Aquino Y Barros L Heleno SA. New frontiers in the exploration of phenolic compounds and other bioactives as natural preservatives. Food Biosci. (2025) 68:106571. doi: 10.1016/j.fbio.2025.106571

[123] Yadav VK Sharma AK Gacem A Pandit J Wany A Kumar A . Emerging trends in the valorization of agricultural waste and their utilization in agricultural, pharmaceuticals, and environmental cleanup. Waste Biomass Valorization. (2025) 16:2779–833. doi: 10.1007/s12649-025-03002-y

[124] Huang Y Chen H Chen W Chen W Zhong Q Pei J . Development and application of polyphenols in food: a comprehensive review. Food Front. (2025) 6:1287–302. doi: 10.1002/fft2.533

[125] Künili IE Akdeniz V Akpinar A Budak SÖ Curiel JA Guzel M . Bioactive compounds in fermented foods: a systematic narrative review. Front Nutr. (2025) 12:1625816. doi: 10.3389/fnut.2025.1625816 40697548 PMC12282486

[126] Bellavite P. Neuroprotective potentials of flavonoids: experimental studies and mechanisms of action. Antioxidants. (2023) 12:280. doi: 10.3390/antiox12020280 36829840 PMC9951959

[127] Chen X Yang J Zhou Y Wang Q Xue S Zhang Y . Research progress and prospects of flavonoids in the treatment of hyperlipidemia: a narrative review. Molecules. (2025) 30:3103. doi: 10.3390/molecules30153103 40807276 PMC12348653

[128] Foata F Duboux S Herzig S Sizzano F Thevenet J Guy P . Identification and biological characterization of a novel NRF2 activator molecule released from the membranes of heat-treated bifidobacterium breve NCC 2950. Mol Nutr Food Res. (2025) 69:e202400770. doi: 10.1002/mnfr.202400770 39911038 PMC12410508

[129] Winter AN Bickford PC. Anthocyanins and their metabolites as therapeutic agents for neurodegenerative disease. Antioxidants. (2019) 8:333. doi: 10.3390/antiox8090333 31443476 PMC6770078

[130] González-Sarrías A Espín-Aguilar JC Romero-Reyes S Puigcerver J Alajarín M Berná J . Main determinants affecting the antiproliferative activity of stilbenes and their gut microbiota metabolites in colon cancer cells: a structure–activity relationship study. Int J Mol Sci. (2022) 23:15102. doi: 10.3390/ijms232315102 36499424 PMC9739882

[131] Feng J Shi Z Ye Z. Effects of metabolites of the lignans enterolactone and enterodiol on osteoblastic differentiation of MG-63 cells. Biol Pharm Bull. (2008) 31:1067–70. doi: 10.1248/bpb.31.1067 18520031

[132] Ribeiro M Alvarenga L Cardozo LFMF Baptista BG Nascimento D Esgalhado M . Urolithin as a metabolite of ellagitannins and ellagic acid from fruits and nuts produced by the gut microbiota: its role on non-communicable diseases. Curr Nutr Rep. (2025) 14:55. doi: 10.1007/s13668-025-00645-0 40180655

[133] Li H Christman LM Li R Gu L. Synergic interactions between polyphenols and gut microbiota in mitigating inflammatory bowel diseases. Food Funct. (2020) 11:4878–91. doi: 10.1039/D0FO00713G 32490857

